# New Species of *Rotundomys* (Cricetinae) from the Late Miocene of Spain and Its Bearing on the Phylogeny of *Cricetulodon* and *Rotundomys*

**DOI:** 10.1371/journal.pone.0112704

**Published:** 2014-11-12

**Authors:** Raquel López-Antoñanzas, Pablo Peláez-Campomanes, Ángeles Álvarez-Sierra

**Affiliations:** 1 School of Earth Sciences, University of Bristol, Bristol, United Kingdom; 2 Departamento de Paleobiología, Museo Nacional de Ciencias Naturales, Consejo Superior de Investigaciones Científicas, Madrid, Spain; 3 Departamento de Paleontología, Facultad de Ciencias Geológicas, Universidad Complutense de Madrid, Departamento de Geología Sedimentaria y Cambio Medioambiental, Instituto de Geociencias IGEO (UCM, CSIC), Madrid, Spain; Team 'Evo-Devo of Vertebrate Dentition', France

## Abstract

The material of *Rotundomys* (Rodentia, Cricetinae) from the Late Miocene fossiliferous complex of Cerro de los Batallones (Madrid, Spain) is described and compared with all species currently placed in the genera *Rotundomys* and *Cricetulodon*. Both the morphology and size variation encompassed in the collection of specimens from Batallones suggest they belong to a single taxon different from the other known species of these genera. A new species *Rotundomys intimus* sp. nov. is, therefore, named for it. A cladistic analysis, which is the first ever published concernig these taxa, has been conducted to clear up the phylogenetic position of the new species. Our results suggest that *Rotundomys intimus* sp. nov. inserts between *R. mundi* and *R. sabatieri* as a relatively primitive taxon inside the clade *Rotundomys*. The new taxon is more derived than *R. mundi* in having a transversal connection between the metalophulid and the anterolophulid on some m1 but more primitive than *R. sabatieri* and the most evolved species of *Rotundomys* (*R. montisrotuni* +*R.bressanus*) in its less developed lophodonty showing distinct cusps, shallower valleys, and the presence of a subdivided anteroloph on the M1. The species of *Cricetulodon* do not form a monophyletic group. As a member of *Rotundomys*, *Rotundomys intimus* sp. nov. is more derived than all of these taxa in its greater lophodonty and the complete loss of the anterior protolophule, mesolophs, and mesolophids.

## Introduction

**Figure 1:**
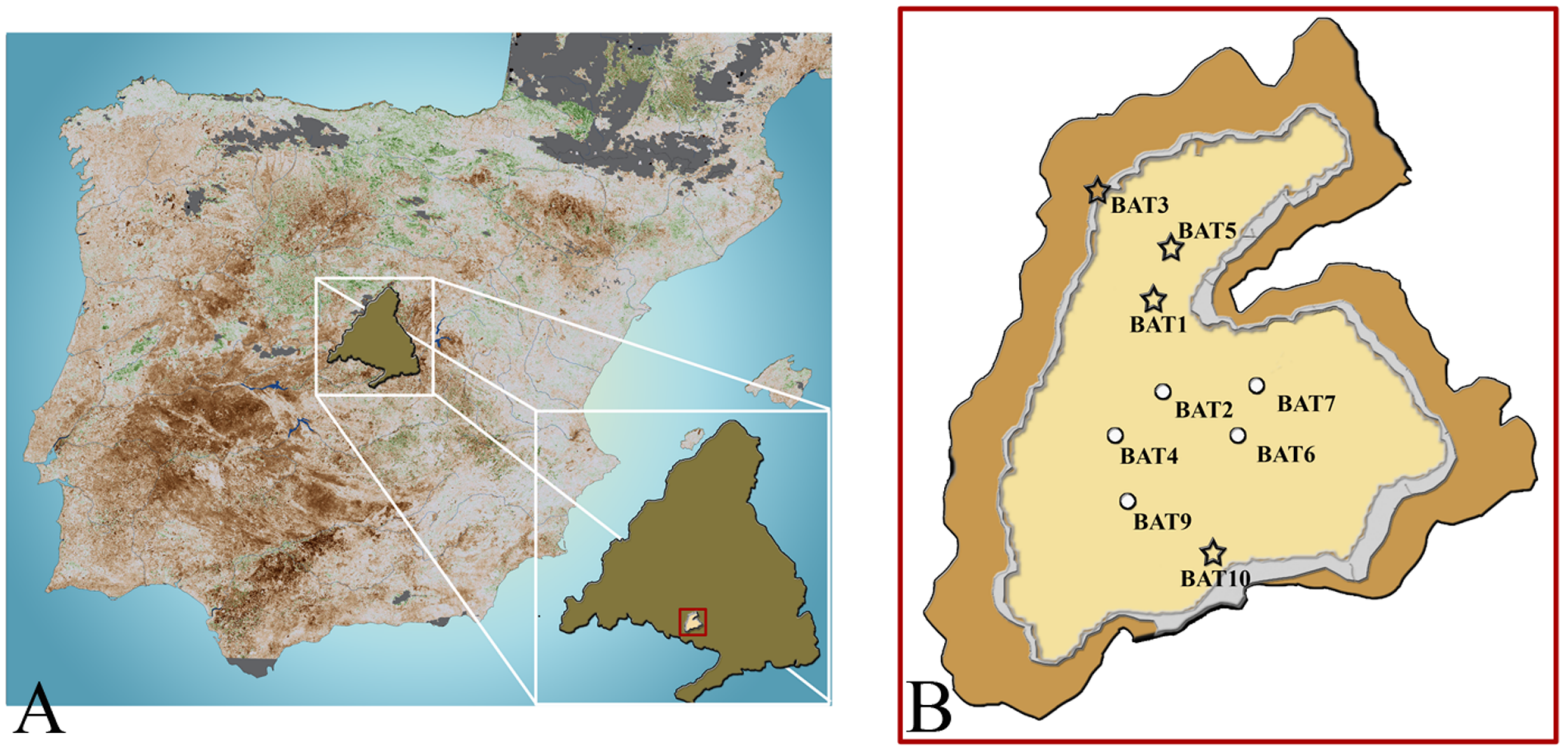
Location of the Cerro de los Batallones fossiliferous complex (Madrid, Spain). (A) location of the Madrid Autonomous Community in the Iberian Peninsula and close-up showing the position of Batallones within it. (B) distribution of the fossiliferous localities at Batallones (those having yielded remains of *Rotundomys* are indicated with a star).

The Cerro de los Batallones fossiliferous complex (CBFC) comprises a set of nine sites that have yielded vertebrate remains of Late Miocene age. It is situated in Torrejón de Velasco, South of the city of Madrid (Spain) (Figure 1). CBFC consists of cavities filled in with clays that are interpreted as having acted as traps for large vertebrates [1].

Besides fishes, amphibians, reptiles, and birds, the sites of Cerro de los Batallones deserve the attention they have received because of both the abundance and pristine conservation of the fossil carnivores and herbivores they yield [2]. However, the micromammals (insectivores, lagomorphs, and rodents) are also represented by numerous and well-preserved remains, although only the cricetodontine *Hispanomys* has been studied in detail so far [3]. The aim of this article is to provide a description and a systematic assessment of the specimens from Batallones assigned to the genus *Rotundomys* and conduct the first cladistic analysis involving not only all the species currently recognized as pertaining to *Rotundomys* but also to the closely related genus *Cricetulodon*. *Rotundomys* is only known with certainty from the Vallesian (late MN9-MN10) of France, Spain, and Portugal. It is characterized by the development of lophodonty and moderate hypsodonty in their cheek teeth. The morphological similarities in the molar crown pattern between the most advanced species of *Rotundomys* and early arvicolids have led to the idea that *Rotundomys* could have been the taxon from which arvicolids were eventually derived [4]. However, a number of other cricetids show arvicoline features so that the exact relationships of *Rotundomys* with respect to arvicolines remains to be determined [5].

## Material and Methods

The material studied herein was collected thanks to numerous summer field and washing campaigns, in which the authors took part. The excavations in the CBFC were carried out according to the authorization issued by the Dirección General de Patrimonio Histórico de la Comunidad de Madrid. All necessary permits were obtained for the described study, which complied with all relevant regulations. We have received permission from the Université Claude Bernard-Lyon 1 (Villeurbanne, France) for the loan of the *Rotundomys* samples that have been used as material of comparison. The acronyms used are: FCA (Fortuna Casa del Acero), FSL (Université Claude Bernard, Villeurbanne, France), ILM (Instituto Lucas Mallada, Madrid, Spain), IPS (Instituto de Paleontología Miguel Crusafont, Sabadell, Spain), MNCN (Museo Nacional de Ciencias Naturales, Madrid, Spain), NMB (Naturhistorisches Museum Basel, Basel, Switzerland), PEC (Pedregueras C), RGM (Naturalis, Leiden, The Netherlands), UNL (Universidade Nova de Lisboa, Lisboa, Portugal), USTL (Université Montpellier 2 Sciences et Techniques, Montpellier, France).

The systematic revision presented below is based on the examination of specimens and casts of the MNCN and FSL collections and data from the literature. We examined dental material of the following taxa:

- *Rotundomys* sp. nov. from Batallones (see below);

- *Rotundomys montisrotundi* from Montredon (Hérault, France) and *Rotundomys* cf. *montisrotundi* from Douvre (Ain, France) (unnumbered specimens);

- *Rotundomys bressanus* from Soblay (Ain, France), Ambérieu 2c, and Ambérieu 1 (Ain, France) (unnumbered specimens);

-casts of *Rotundomys* cf. *mundi* from Terrasa (Barcelona, Spain) (unnumbered specimens) and *R. freiriensis* from Freiria do Rio Maior (Santarém, Portugal) (unnumbered specimens).

**Figure 2:**
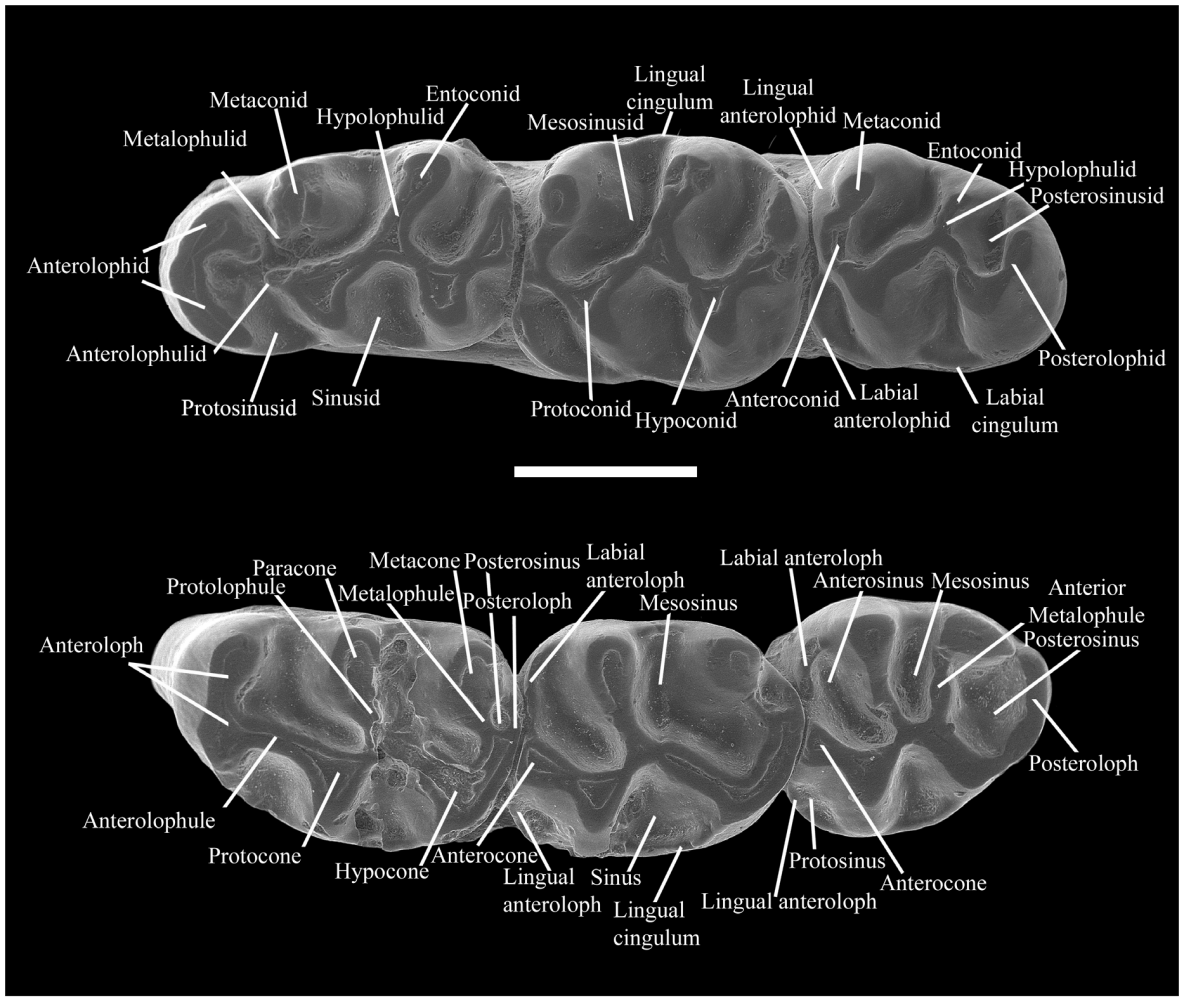
Dental terminology used in this work.

The new specimens have been described and compared with the equivalent teeth of all the species of *Rotundomys* known to date and some *Cricetulodon*. First, second, and third lower molars are designated as m1, m2, and m3, respectively, and first, second, and third upper molars as M1, M2, and M3. The terminology used in the tooth descriptions follows the rodent dental terminology of Freudenthal et al. [6] with some adjustments (see Figure 2). The occlusal measurements (greatest length and greatest width; Table 1) of the teeth of *Rotundomys* from Batallones have been obtained with a Nikon digital counter CM-6S measuring device. The calculations of the statistical descriptives and Analyses of Variance (ANOVA) have been carried out with a standard software (SPSS Statistics version 18.0, SPSS Inc., Chicago, IL, USA). Tests on normality and homogeneity of variance have been performed with this software before the Analyses of the Variance. The relative reduction in the length of the third molars was calculated using the (mean Length of M1)/(mean Length of M3) and (mean Length m1)/(mean Length m3) ratios, which is a classic method for evaluating the degree of reduction of the third molars [3]. For *Rotundomys* samples whose variance was known, the standard error of the ratio (SER) was calculated using the Delta approximation (sensu Ratio technique in SPSS) [3]. The formula used is:

**Table 1:** Length and width measurements (mm) of the lower and upper molars of *Rotundomys intimus* sp. nov. from Batallones (Madrid, Spain).

		Length					Width			
		N	Min.	Mean	Max.	s.d.	Min.	Mean	Max.	s.d.
**m1**	Batallones 3	4	2.09	2.18	2.25		1.16	1.22	1.34	
	Batallones 5	13	1.92	2.02	2.14	0.0676	1.18	1.26	1.48	0.0780
**m2**	Batallones 1	1		1.77				1.44		
	Batallones 3	5	1.67	1.77	1.87	0.0891	1.30	1.41	1.52	0.0788
	Batallones 5	16	1.57	1.71	1.79	0.0650	1.32	1.41	1.51	0.0553
**m3**	Batallones 1	1		1.74				1.30		
	Batallones 3	4	1.50	1.64	1.78		1.24	1.37	1.44	
	Batallones 5	12	1.39	1.55	1.73	0.1036	1.23	1.30	1.36	0.0438
**M1**	Batallones 10	2	1.89		1.97		1.37		1.38	
	Batallones 3	2	2.12		2.15		1.55		1.60	
	Batallones 5	4	2.03	2.07	2.15		1.32	1.39	1.51	
**M2**	Batallones 1	1		1.75				1.37		
	Batallones 10	2	1.72		1.80		1.37		1.45	
	Batallones 3	2	1.69		1.71		1.53		1.55	
	Batallones 5	4	1.64	1.72	1.82		1.36	1.42	1.51	
**M3**	Batallones 1	1		1.62				1.35		
	Batallones 10	2	1.49		1.51		1.30		1.32	
	Batallones 3	2	1.20		1.42		1.27		1.36	
	Batallones 5	5	1.35	1.50	1.70	0.1302	1.31	1.37	1.46	0.0695



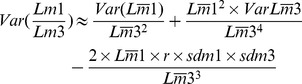
 (where Var is the variance, r the coefficient of correlation between the length of the first and third molars, and sd is the standard deviation)

The coefficient of correlation of all the *Rotundomys* and *Cricetulodon* samples included in Table 2 is 0.503 for the upper molars and 0.527 for the lower ones.

**Table 2:** m1/m3 and M1/M3 (mm) length ratio for all species of *Rotundomys* and *Cricetulodon* known to date from various localities.

Taxa	Locality	Age	Lm1/Lm3	SER	LM1/M3	SER
*Democricetodon franconicus*	Erkertshofen 1	MN4	(200/150) 1.29		(200/146) 1.82	
*Cricetulodon hartenbergeri*	Pedregueras 2C	MN9 (I)	(48/76) 1.19		(58/69) 1.55	
*Cricetulodon sabadellensis*	Can Llobateres	MN9 (I)	(?/?) 1.28		(?/?) 1.55	
*Cricetulodon bugesiensis*	Soblay	MN10 (J2)	(16/12) 1.18	0.065	(14/10) 1.57	0.094
*Cricetulodon meini*	Casa del Acero	MN12	(3/1) 1.31	0.016	(6/6) 1.65	0.047
*Cricetulodon lucentensis*	Crevillente 17	MN12/MN13	(5/5) 1.57	0.075	(7/4) 1.89	0.170
*Cricetulodon lucentensis*	Crevillente 8	MN12/MN13	(3/1) 1.51		(9/4) 1.97	0.058
*Rotundomys bressanus*	Soblay	MN10 (J2)	(6/8) 1.15		(11/3) 1.45	
*Rotundomys mundi*	Hijar	MN10 (J1)	(1/1) 1.29		(0/1)	
*Rotundomys sabatieri*	Lo Fournas 16M	MN10 (J1)	(56/51) 1.23	0.052	(53/58) 1.48	0.072
*Rotundomys sabatieri*	Lo Fournas 6C	MN10 (J1)	(27/20) 1.22	0.057	(32/21) 1.45	0.090
*Rotundomys montisrotundi*	Lo Fournas 7	MN10 (J1)	(26/50) 1.23	0.053	(19/-) -	-
*Rotundomys montisrotundi*	Montredon	MN10 (J1)	(98/96) 1.22	0.056	(86/76) 1.43	0.072
*Rotundomys freiriensis*	Freiria de Rio maior	MN10 (J1)	(4/4) 1.31	0.085	(2/4)	
*Rotundomys intimus* sp.nov.	Batallones 5	MN10 (J2)	(13/12) 1.31	0.074	(4/5)1.38	0.108
*Rotundomys intimus* sp.nov.	Batallones 3	MN10 (J2)	(4/4) 1.33	0.080	(2/2)1.63	0.187

Calculated from data in [7], [15], [17], [30], [31] and Aguilar (personal communication). SER: Standard Error of the Ratio.

The cladistic analysis carried out in this work treated as ingroup all known species of the genera *Cricetulodon* and *Rotundomys*. Therefore, the taxonomic units are: *Cricetulodon hartenbergeri*, *C. sabadellensis*, *C. bugesiensis*, *C. meini*, *C. lucentensis*, *Rotundomys montisrotundi*, *R. bressanus*, *R. mundi*, *R. sabatieri*, *R. freiriensis*, *Rotundomys* sp. nov. from Batallones. *Democricetodon franconicus* has been selected as outgroup. It is a well-known species of *Democricetodon*, which is a genus from which *Cricetulodon* is supposed to have been derived (see e.g., [7]). A total of 42 phylogenetically informative characters (mainly of dental morphology) have been coded (Text S1). 31 characters are binary, whereas 11 are multistate. Owing to the lack of a priori information, all characters were unordered and equally weighted (Fitch optimality criterion). As some species are known so far from only a few specimens, the influence of intraspecific variation in the scoring of the characters could not be assessed.

The data matrix (Text S2) was built using Mesquite version 2.6 (Maddison WP & Maddison DR, Mesquite Project, Vancouver, Canada) and processed with TNT [8] with the "implicit enumeration" option. Branch support was estimated through two complementary indices: Bremer support [9] and relative Bremer support [10].

### Nomenclatural acts

The electronic edition of this article conforms to the requirements of the amended International Code of Zoological Nomenclature, and hence the new name contained herein is available under that Code from the electronic edition of this article. This published work and the nomenclatural acts it contains have been registered in ZooBank, the online registration system for the ICZN. The ZooBank LSIDs (Life Science Identifiers) can be resolved and the associated information viewed through any standard web browser by appending the LSID to the prefix "http://zoobank.org/". The LSID for this publication is: urn: lsid: zoobank.org: pub: 308BFD06-6024-4BF5-9F0C-F9C6415F8201. The electronic edition of this work was published in a journal with an ISSN, and has been archived and is available from the following digital repositories: PubMed Central, LOCKSS.

## Results

Order rodentia Bowdich, 1821 [11]

Family cricetidae Fischer 1817 [12] (as Cricetini)

Subfamily cricetinae Fischer 1817 [12] (by the principle of coordination)

Genus rotundomys Mein, 1965 [13]

Type species: *Rotundomys montisrotundi* (Schaub, 1944) [14]

Assigned species: *Rotundomys bressanus* Mein, 1975 [15]; *Rotundomys mundi* Calvo, Elizaga, López-Martínez, Robles et Usera, 1978 [16]; *Rotundomys freiriensis* Antunes et Mein, 1979 [17]; *Rotundomys sabatieri* Aguilar, Michaux et Lazzari, 2007 [18]

**Figure 3:**
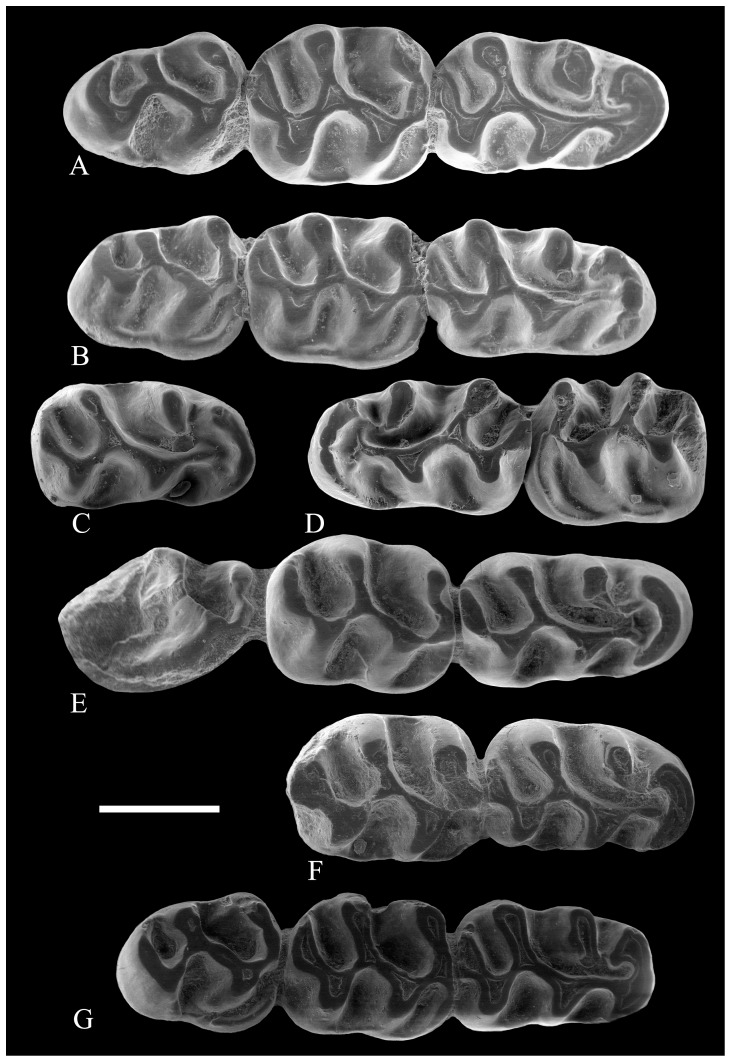
Lower molars of *Rotundomys intimus* sp. nov. (A) right mandible with m1–m3 (BAT5'10-07); (B) right mandible with m1–m3 (BAT5'11-01); (C) right m1 (BAT5'2006-I16d-02); (D) left mandibular fragment with m1-m2 (BAT5'2006-I16c-04); (E) right mandible with m1–m3 (BAT5'10-09); (F) right mandibular fragment with m1–m2 (BAT5'10-04); (G) right mandible with m1–m3 (BAT5'06-H14-120). Scale bar  = 1 mm.

**Figure 4:**
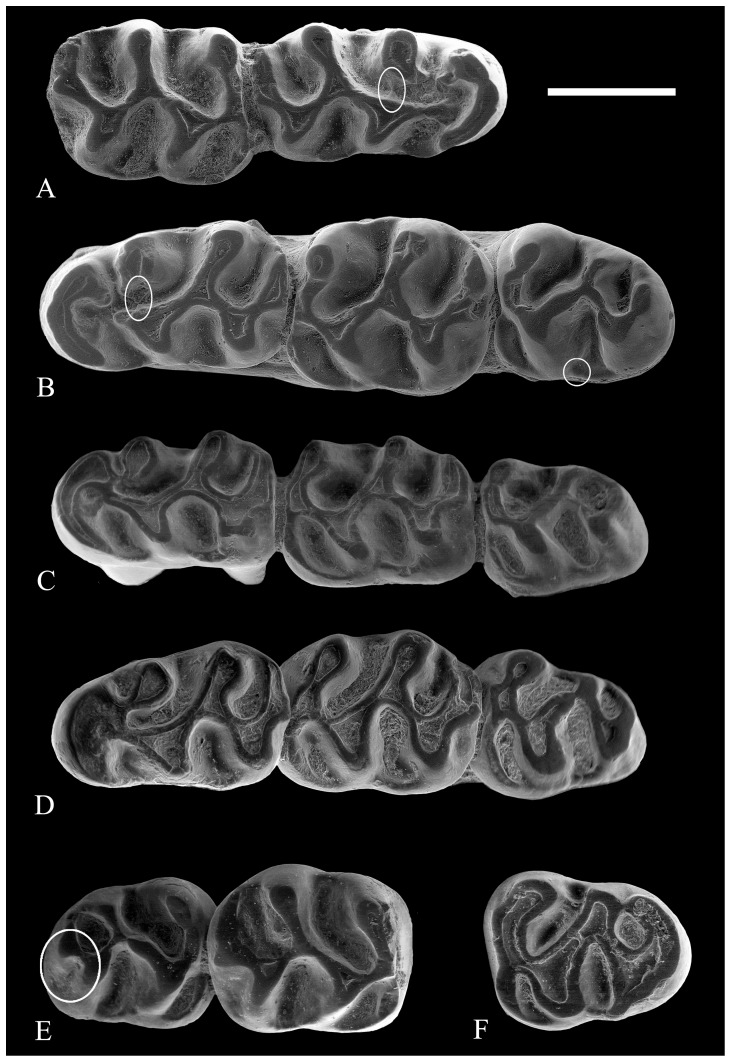
Lower molars of *Rotundomys intimus* sp. nov. (A) right mandibular fragment with m1–m2 (BAT 5'06-I15-29?24?), transverse connexion between the anterolophulid and the metalophid circled; (B) left mandible with m1–m3 (BAT5-2006-I15-5); (C) left mandible with m1–m3 (BAT5'10-06), transverse connexion between the anterolophulid and the metalophid on the m1 and cuspule on the m3 circled; (D) left mandible with m1–m3 (BAT5'10-15); (E) right mandibular fragment with m2–m3 (BAT5'10-05), labial posterolophid circled; (F) left m3 (BAT5'10-12). Scale bar  =  mm.

**Figure 5:**
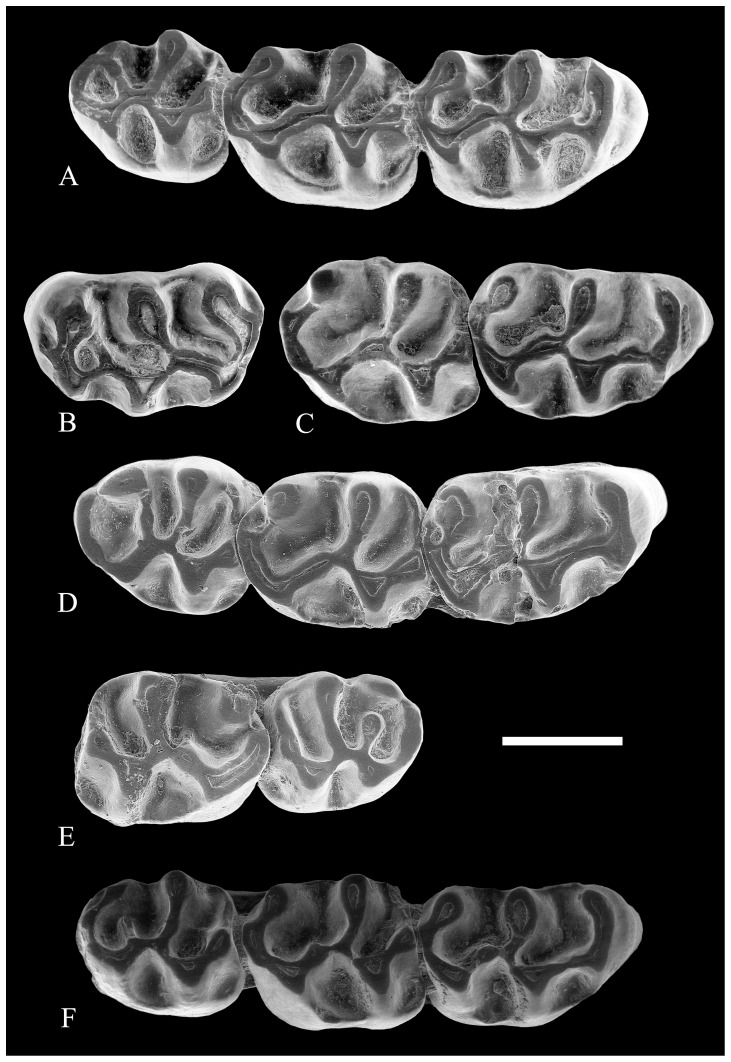
Upper molars of *Rotundomys intimus* sp. nov. (A) right maxilla with M1-M3 (BAT5'11-02); (B) left M1 (BAT5'10-14); (C) right maxillary fragment with M1–M2 (BAT5'06-I15-28); (D) right maxilla with M1–M3 (BAT5'06-01); (E) left maxillary fragment with M2-M3 (BAT5'06-I15-16); (F) right maxilla with M1–M3 (BAT 5'10-01). Scale bar  = 1 mm.

**Figure 6:**
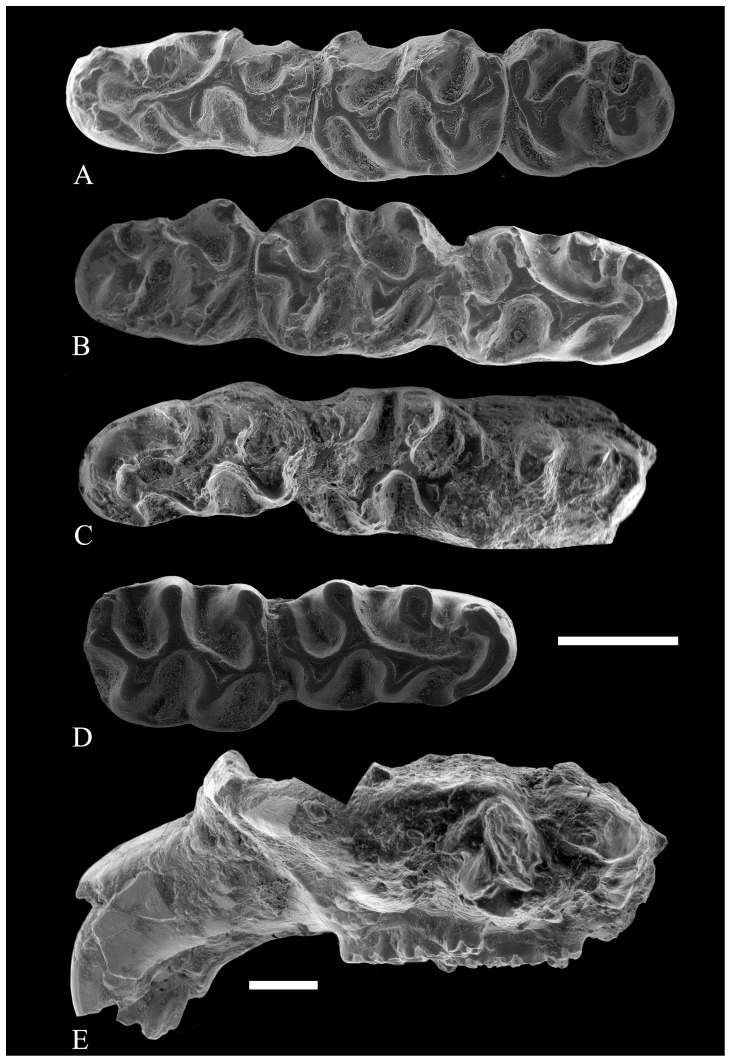
Lower molars of *Rotundomys intimus* sp. nov. (A) left mandible with m1–m3 (BAT3'07-234); (B) right mandible with m1–m3 (BAT3'08-1); (C) left mandible with m1–m3 (BAT3-09); (D) right mandible fragment with m1–m2 (BAT3-12). **Cranium:** (E) lateral view (BAT3'06-758). Scale bar  = 1 mm.

**Figure 7:**
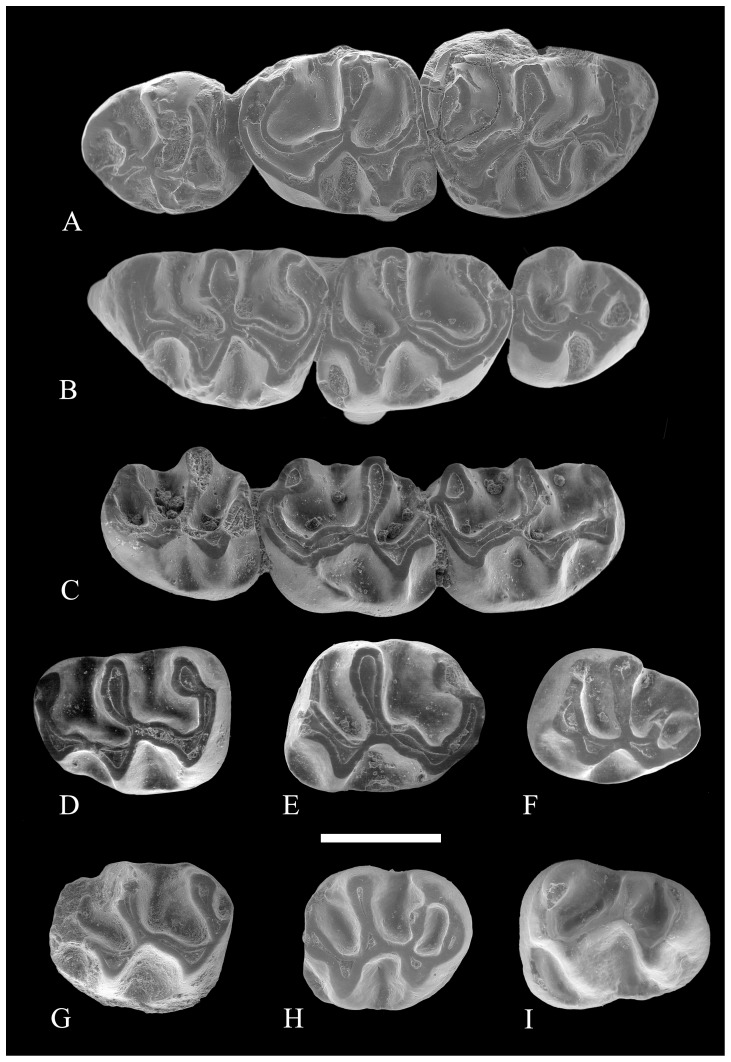
Upper molars of *Rotundomys intimus* sp. nov. (A) right maxilla with M1–M3 (BAT3'06-758); (B) left maxilla with M1–M3 (BAT3'06-758); (C) right maxilla with M1–M3 (BAT10'09-G5); (D) left M1 (BAT10'09-G5); (E) left M2 (BAT10'09-G5); (F) left M3 (BAT10'09-G5); (G) left M2 (BAT1'01-D4); (H) left M3 (BAT'91-02). **Lower molars:** (I) left m3 (BAT1-05). Scale bar  = 1 mm.

*Rotundomys intimus* sp. nov. urn: lsid: zoobank.org: act: 9BA54135-D98E-4047-88EF-7494ACA713CF (Figures 3, 4, 5, 6, 7)

Etymology: From the Latin *intimus*, the most interior, in reference to the fact that the *locus typicus* is situated in the innermost position with respect to the other Spanish sites, which are much closer to the shore.

Holotype: Hemimandible with m1–m3: BAT5'10-07.

Paratype: Hemimandibles with m1–m3: BAT5'10-09, BAT5'11-01, BAT5'06-I15-5, BAT5'06-H14-120, BAT5'10-06, BAT5'10-07; mandibular fragments with m1–m3 (m1 broken): BAT5'10-11, BAT5'10-02; mandibular fragment with m1: BAT5-2006-I16d-02; mandibular fragments with m1–m2: BAT5'10-10, BAT5-2006-I16c-04, BAT5-2006-I15-29?24?, BAT5'10-04; mandibular fragments with m2-m3: BAT5'10-05; BAT5'10-03; mandibular fragment without teeth: BAT5'06-I15-17; isolated m3 BAT5'10-12; isolated m2 BAT5'10-08; maxillae with M1–M3: BAT5-2006-SIN UBICAR; BAT5'10-01; BAT5'11-02; maxillary fragments with M1: BAT5'10-14; maxillary fragments with M2–M3: BAT5-2006-I15-16; maxillary fragments with M1–M2: BAT5'06-I15-28; isolated M3: BAT5'10-13.

Referred material: Hemimandibles with m1–m3: BAT3'07-234, BAT3-12, BAT3-09; isolated m2: BAT1991-03; isolated m3: BAT1-05; fragmentary skull with complete maxillae: BAT3-2006-758; maxillae with M1–M3: BAT10'09-G5; isolated M2: BAT1-2001-D4; isolated M3: BAT1991-02.

Repository institution: MNCN-CSIC collections.

Type locality: Batallones 5, Torrejón de Velasco, Madrid, Spain.

Age: MN10, Late Vallesian, Late Miocene.

Other localities: Batallones 1, Batallones 3, and Batallones 10, Torrejón de Velasco, Madrid, Spain.

Diagnosis: Cricetinae with lophodont cheek teeth; protoconid connected to the hypolophulid in a regularly curved crest on the first lower molars; wide valleys usually closed by thin, low cingula; weak anterior connections; anterior protolophule and mesolophs/ids absent and anterior metalophule usually absent; strongly fordwardly-directed anterior metalophulid and strongly backwardly-directed posterior metalophule. Well-developed metacone on the M3.

Differential diagnosis: Differing from the species of *Cricetulodon* in being more lophodont, lacking the anterior protolophule, and usually the anterior metalophule and the mesolophs/ids, in having the protoconid connected to the hypolophulid in a regularly curved crest on the m1 and the M3 much less reduced. Differing from *Rotundomys bressanus* in being smaller, less lophodont, with distinct cusps/ids, better developed cingula surrounding the valleys and having low, weak and interrupted metalophulid and anterolophulid on the m1. Differing from *R. mundi* in being larger, lacking the anterior metalophule on the M2, and in having the M3 less reduced and without strong connection between paracone and labial anteroloph. Differing from *R. montisrotundi* and *R. sabatieri* in being smaller, less lophodont, and in having more distinct cusps/ids and shallower valleys. Differing from *R. freiriensis* in having the anterolophulid, a strongly forwardly-directed metalophulid on the m1, lingual anterolophid on the m2, and the M3 much less reduced.

### Description

#### Material from the type locality (Batallones 5)

m1: The teeth are elongated, being widest at the level of the hypoconid. The anterior part of the teeth is fairly broad. The anterolophid, which is as high as the main cusps, is usually divided in two or three cuspids, but it may consist of a single ridge. The labial anterolophid descends and nearly joins the protoconid, enclosing a wide valley (protosinusid). The poorly developed metalophulid points strongly forwards, being as it is almost longitudinal. It does not usually connect to the anterolophid and, when it does, this connexion is thin. The mesolophid is absent. The protoconid joins the hypolophulid in a regularly curved crest. The protoconid and the hypoconid have about the same size. The mesosinusid is large, curved, and partially closed in most specimens by a thin and low lingual cingulum ridge. The posterolophid bulges as a posteroconid; from it, a lingual crest descends, but does not usually reach the entoconid. Thus, the posterosinusid is not completely closed. The sinusid is nearly transverse and it is closed by a low labial cingulum ridge.

Three out of 11 specimens (BAT5'10-07, BAT5'2006-I16d-02 and BAT5'11-01; Figure 3A–C) have the metaconid isolated and the protoconid joined with the labial or central part of the anterolophid through a long longitudinal anterolophulid. 2 out of 11 (BAT5'10-09 and BAT5'2006-I16c-04; Figure 3E–D) have the metaconid connected to the lingual anterolophid through a weak metalophulid that strongly points forwards, the protoconid joins the labial anterolophid through a long anterolophulid, and the anterolophulid and the metalophulid are transversely connected. 3 out of 11 specimens (BAT5'10-04, BAT 5'06-I15-29?24?, BAT5'06-H14-120; Figures 3F–G, 4A) have similar morphology, but they lack the connexion between the anterolophulid and the metalophid.

Three out of 11 specimens (BAT5-2006-I15-5, BAT5'10-06 and BAT5'10-15; Figure 4B–D) have the anterolophulid connected to the lingual anterolophid. Interestingly enough, BAT 5'06-I15-29?24? and BAT5-2006-I15-5 (Figure 4A–B) show an additional, thin and transversal connexion in between metaconid and protoconid (anterior metalophulid). These teeth are two rooted.

m2: The maximal width of the tooth is at the level of the hypoconid. The anteroconid is distinct and centrally located; from it, a strong labial anterolophid runs down, reaches the protoconid, and closes the protosinusid. The lingual anterolophid is absent in the entire sample. The metalophulid runs obliquely forwards and the mesolophid is absent. As in the m1, the protoconid and the entoconid form a continuous arch. The labial cusps have nearly the same size. The mesosinusid is large and curved; it is closed by a strong and low lingual cingulum ridge. The posterolophid is bulged in a posteroconid; from it, runs a lingual crest that joins the entoconid and closes the posterolingual sinusid. The nearly transverse sinusid is closed by a low and strong labial cingulum ridge. These teeth have two roots.

m3: Except for BAT5'10-05, BAT5'10-07, BAT5'10-15, BAT5'10-06 (Figures 3A, 4C–E) and possibly a worn specimen (BAT5'10-12; Figure 4F), which have the posterolophid connected to the entoconid closing the posterolingual sinusid, the remainining m3 from Batallones 5 show a short posterolophid that does not join with the entoconid. All m3 are somewhat posteriorly reduced and, therefore, their hypoconid is reduced as well. The anteroconid is large and slightly lingually located. They show a low and strong labial anterolophid that connects to the anterior wall of the protoconid, closing the protosinusid. The lingual anterolophid is lacking. Due to the very anterior position of the metalophulid, the lingual anterior cingulum is absent. The posterior arm of the protoconid is very long and it connects to the hypolophulid, but there is no longer the regularly curved crest that characterized the m1 and m2 of this taxon. The mesosinusid is large and it is closed by a thin low lingual cingulum ridge. The sinusid is closed by a strong labial cingulum ridge with a cuspule on the posterior wall of the protoconid in some specimens (BAT5'06-I15-5; Figure 4B). Some specimens (BAT5'10-05; Figure 4E) show a short labial posterolophid that closes the small labial posterosinusid. These teeth are two rooted.

M1: The prelobe (all structures anterior to the protocone and paracone) is long and the posterior side of the protocone is located at about the midpoint of the teeth. The anterolophule connects the protocone lingually with the anteroloph, which is usually divided into two anterocones. 2 out of 4 specimens (BAT5'11-02 and BAT5'10-14; Fig 5A–B) show a labial spur on it that connects to the anteroloph. Another specimen (BAT5'06-I15-28; Figure 5C) shows at this level a slight inflation that may correspond to this spur and BAT5'06-01 (Figure 5D) lacks all trace of it. None of the teeth have a true mesoloph but they have the anterior arm of the hypocone somewhat inflated. All the teeth but BAT5'11-02 (Figure 5A), which has a low and thin anterior metalophule, lack this structure. The anterior protolophule is absent in all specimens. The posterior protolophule and metalophule are posterolabially directed. The metacone is located on the posterolabial corner of the tooth and it is connected to the posteroloph through the posterior metalophule.

Two specimens (BAT5'06-01 and BAT5'10-14; Figure 5B, D) show a thin labial ridge emerging from the end of the posteroloph, enclosing a small labial posterosinus. The sinus is transverse. Thin and low labial and lingual cingula enclose the valleys. These teeth are three rooted (the lingual root is the largest).

M2: The M2 from Batallones 5 are widest at the level of the paracone. They have a large anterocone, slightly lingually located. The labial and lingual anteroloph are low but well developed. The lingual anteroloph, placed much lower than the labial one, joins with the protocone and closes the protosinus. The labial anteroloph does not usually reach the paracone. A strong labial cingulum ridge closes the mesosinus. Except for specimen BAT5'06-I15-16 (Figure 5E), in which a very thin and short mesoloph is noticed, a true mesoloph is absent in the entire sample. However, the hypocone, which forms a wider V than the protocone, has its anterior arm slightly inflated at the level of the mesoloph. The metacone is located on the posterolabial edge of the teeth. These teeth lack anterior protolophule and metalophule. The posterior protolophule is slightly oblique, whereas the posterior metalophule points strongly backwards, joining with the posteroloph. In 2 out of 5 specimens (BAT5'06-01 and BAT5'06-I15-16; Figure 5D–E), the end of the posteroloph extends as a thin labial ridge that runs posterolabially and reaches the posterior wall of the metacone, enclosing a very small posterosinus. In the remaining specimens, the posterosinus is lacking. All specimens have a transverse sinus, which is closed by a low and distinct lingual cingulum. The labial valleys are closed by thin but distinct low cingula. These teeth have four roots.

M3: The posterior portion is somewhat reduced, so the hypocone, even though it is well developed, is smaller than the protocone. The anterocone is large and located slightly lingually. The labial anteroloph is higher and better developed than the lingual one, which is very low but distinctly noticeable. They join with the protocone and paracone, respectively, closing as they do the anterior valleys. All specimens show the anterior metalophule, which is generally long and reaches the metacone, but it can also be of medium length and free (as in BAT5'10-01 and BAT 5'06-I15-16; Figure 5E–F). The posterior metalophule is fused with the posteroloph. All M3 from Batallones 5 have the posteroloph connected to the posterior wall of the large metacone. The lingual cingulum is thin and low and closes the transverse sinus. The labial one is much less developed than it is on the M1 and M2 of this taxon. These teeth are three rooted (a single lingual and a double root or three separate roots).

#### Material from the other localities

Batallones 3: The material from this locality is composed of 5 hemimandibles, one of them without m1, and a cranium. The morphology of the material from Batallones 3 is similar to that described of the population of Batallones 5. Nevertheless, there are several morphometrical differences. Except for specimen BAT3-12, the m1 are longer and narrower than those of Batallones 5. The scarcity of the material from Batallones 3 does not allow for precise statistical testing of the metrical differences between those assemblages for most dental elements. The tests carried out on samples larger than 4 specimens reveal no significant differences between Batallones 3 and 5 except for the greater length of the m1 (t-student  = 3.77, signification (bilateral)  = 0.002) in Batallones 3. Table 2 shows that the ratio between M1 and M3 length is higher in Batallones 3 than in Batallones 5 implying a possible trend towards relatively smaller M3, not observed on theoretically more advanced forms of *Rotundomys*.

The morphotypes of the m1 observed in Batallones 3 correspond to some found at Batallones 5. For instance, BAT3'07-234 (Figure 6A) has the metaconid connected to the lingual anterolophid through a weak metalophulid that strongly points forwards and the protoconid is connected to the labial anterolophid through a long anterolophulid. BAT3'08-1 (Figure 6B) has the metaconid isolated and the protoconid joined with the labial or central part of the anterolophid through a long longitudinal anterolophulid. Besides, BAT3-09 (Figure 6C) and BAT3'07-234 (Figure 6A) have the metaconid connected to the protoconid by a thin transverse ridge. The former has the metaconid isolated from the anterolophid. The m2 and m3 from Batallones 3 do not differ from those of Batallones 5. The particular morphology shown by the specimens BAT3-12 (Figure 6D) and BAT3-2007-234 (Figure 6A), which have a small labial posterolophid, is found also in some specimens from Batallones 5 (BAT5'10-05; Figure 4E).

With regard to the upper molars, their morphology is also similar to the specimens from Batallones 5 that lack the spur of the anterolophule. As the teeth of the single specimen (Figures 6E, 7A–B) we have from this locality are worn, it is not possible to discern if they had an additional ridge arising from the end of the posteroloph.

Batallones 10 locality: From this locality only two maxillary fragments with M1-M3 belonging to the same individual have been recorded (Figure 7C–F). The morphology of these teeth is similar to that found in the population of Batallones 5. In particular, the M1 match well those from Batallone 5 that lack the spur of the anterolophule.

Batallones 1 locality: This locality has yielded three isolated teeth: 1M2, 1M3, and 1 m3 (Figure 7G–I). The M2 and M3 are similar in morphology to those of the population of Batallones 5 and fall within its size range. The morphology of the m3 is also similar but it is slightly larger than those of the type population.

### Comparisons

#### Comparison with *Cricetulodon hartenbergeri* (Freudenthal, 1967) [19]

This species was originally coined by Freudenthal [19] as belonging to the genus *Rotundomys*. Bruijn et al. [20] and subsequent authors reallocated it to the genus *Cricetulodon*. The holotype of this species (PEC 585) is a first lower molar from the late MN9 locality of Pedregueras IIC, IIA (Zaragoza, Spain), which is housed in IPS. Additional material from this taxon has been recovered from the MN9 localities of La Roma·3, Peralejos 5 (Teruel, Spain) [21], Santiga, Can Ponsic, Can Petit, Autopista de Rubí-Terrasa 6E and Viladecavalls (Barcelona, Spain) [22], [23], [24], [25], Ampudia 9, Torremormojón, 4, 3, 2 (Palencia, Spain) and Tordehumos 3 (Valladolid) [26], [27], [28], [29] as well as from the MN10 localities of La Roma 6, 7, 11, and Puente Minero 2 (Teruel, Spain) [21].

The cheek teeth of this species are smaller than the equivalent teeth of *Rotundomys intimus* sp. nov. from Batallones. In addition, this species is characterized by the presence of mesoloph and anterior protolophule on the upper molars. Moreover, the M3 of *Cricetulodon hartenbergeri* are much more reduced than those of *R. intimus* sp. nov. and their morphology is very different. For instance, the metacone of the former taxon is very small and may even disappear as a distinct cusp by fusion with the posteroloph. In contrast, the upper molars of *R. intimus* sp. nov. lack the mesoloph and the anterior protolophule and their M3 are less reduced and characterized by a well-developed metacone. With regard to the lower molars, most *C. hartenbergeri* have a mesolophid, which is absent in *R. intimus* sp. nov. In addition, most m1 of *C. hartenbergeri* have the anterolophulid connected to the lingual cusp of the anterolophid, whereas only 20% of the m1 of *R. intimus* sp. nov. show this kind of connection. Finally, in the m1 and m2 of *C. hartenbergeri*, the protoconid does not join the hypolophulid in a regularly curved crest as is the case in *R. intimus* sp. nov.

#### Comparison with *Cricetulodon sabadellensis* Hartenberger, 1965 [30]

The holotype of this species is a maxillary fragment with M1-M2 (CL1392) from the MN9 site of Can Llobateres 1, which is housed in IPS [30]. Additional material of this taxon has been reported from Can Pallars de Llobateres 3 [24], Can Coromines 2, Autopista de Rubí-Terrasa 3B, Autopista de Rubí-Terrasa 8, Viladecavalls, Torrent de Febulines M, and Can Purull. (Barcelona, Spain) [23], [7].

Most of the M1 and M2 of *Cricetulodon sabadellensis* have a short mesoloph and a labial spur of the anterolophule directed towards the paracone (anterior protolophule). These structures are lacking in almost all the M1 and M2 of *Rotundomys intimus* sp. nov. The M3 of *C. sabadellensis* also have double protolophule and a short anterior metalophule. In contrast, the M3 of *R. intimus* sp. nov. lack the anterior protolophule and have a longer anterior metalophule. The morphology of the m1 of *C. sabadellensis* is very different from that of *R. intimus* sp. nov. The latter species has the metalophulid nearly longitudinal, whereas it is transverse in *C. sabadellensis*.

#### Comparison with *Cricetulodon bugesiensis* Freudenthal, Mein et Martín-Suarez, 1998 [7]

The holotype (FSL 65897) of this species is a left isolated m1 from the MN10 locality of Soblay (Ain, France) [7], which is housed in FSL. Additional material of this species has been recovered from the MN10 localities of Douvre (Ain, France) and Dionay (Isère, France) as well as from the MN 11 localities of Crevillente 2 (Alicante, Spain) [7].

About half the M1 of *Cricetulodon bugesiensis* have double protolophule and anterior metalophule and almost all of them have the mesoloph, usually of medium length or long. In contrast, the M1 of *Rotundomys intimus* sp. nov. lack a true mesoloph and the anterior protoloph and metaloph. All the M2 of *C. bugesiensis* have a double protoloph, most of them a true mesoloph, and about half the specimens show an anterior metalophule that can be formed by the mesoloph or be independent from it. In contrast, all the M2 of *R. intimus* sp. nov. lack the anterior protolophule and metalophule and only one specimen shows a very short mesoloph. The M3 of *C. bugesiensis* also have a double protoloph and a number of them have a mesoloph, which is absent in *R. intimus* sp. nov. With regard to the lower molars, over half of the m1 and some m2 and m3 have a mesolophid, which is absent on all lower molars of *R. intimus* sp. nov.

#### Comparison with *Cricetulodon meini* (Agustí, 1986) [31]

This species was originally created by Agustí [31] as belonging to the genus *Kowalskia*. Later, this taxon was reallocated to the genus *Cricetulodon* [7] on the basis of the lingual anterolophulid on the m1, a reduced M3, and reduced mesolophs and mesolophids. The holotype (FCA-237), a right isolated M1 from the MN12 locality of Casa del Acero (Murcia, Spain), is housed in IPS.

The upper molars of *Cricetulodon meini* have a double protolophule and they can bear a mesoloph. In contrast, those of *Rotundomys intimus* sp. nov. lack the anterior protolophule and the mesoloph. Moreover, the M3 of *C. meini* are much more reduced than those of *R. intimus* sp. nov. and lack the lingual anteroloph, which is well developed in the latter species. The m1 of *C. meini* have a metalophulid that does not point strongly forwards whereas that in *R. intimus* sp. nov. is nearly longitudinal. In addition, the lower molars of the former taxon usually have the mesolophid, which is lacking on those of *R. intimus* sp. nov.

#### Comparison with *Cricetulodon lucentensis* (Freudenthal, Lacomba et Martín-Suarez, 1991) [32]

This species was originally attributed to the genus *Neocricetodon* by Freudenthal et al. [32]. Subsequently, Freudenthal et al. [7] transferred it to the genus *Cricetulodon* on the basis of the clearly lingual anterolophulid of some m1 and the strong reduction of the third molars. The holotype of this taxon (RGM 404 677) is a right m1 from the MN12 locality of Crevillente 17 (Alicante, Spain) [32] that is housed in RGM. Additional material of this species has been recovered from Crevillente 5 and Crevillente 8 (Alicante, Spain) [32].

Some m1 of *Cricetulodon lucentensis* have a long mesolophid, which is absent on the equivalent teeth of *Rotundomys intimus* sp. nov. In addition, the m1 of the former species have the metalophulid directed much less forwards than what can be observed in the latter. The m3 of *C. lucentensis* are much more reduced than those belonging to *R. intimus* sp. nov. In addition, the m3 of the former taxon have a lingual anterolophid that is absent in the latter species. Most of the M1 and M2 of *C. lucentensis* have a double protolophule, a double or anterior metalophule, and a mesoloph. In contrast, the M1 and M2 of *R. intimus* sp. nov. lack the anterior protolophule, the anterior metalophule, and the mesoloph. In addition, the M2 of *C. lucentensis* have a well-developed labial anteroloph that closes a large anterosinus. The M3 of *C. lucentensis* are morphologically very different and much more reduced than those of *R. intimus* sp. nov.

#### Comparison with *Rotundomys bressanus* Mein, 1975 [15]

This species was erected on the basis of 43 isolated cheek teeth from the late MN10 locality of Soblay (Ain, France) [15]. After the study of additional material from Montredon (Herault, France), Aguilar [33] considered this taxon a synonym of *Rotundomys montisrotundi*. However, Freudenthal et al. [7] argued that there were enough characters to distinguish the two taxa (for instance, the overall size, the wear surface of protoconid and protocone, the degree of reduction of both, labial anterolophid on the m1 and posterolophid-entoconid connection on the m3) and, therefore, they considered *R. bressanus* a valid species, an opinion with which we concur.

The holotype (FSL 65443) of this species is an isolated left M1 housed in FSL.

Additional material of this species has been recovered from the late MN10 sites of Ambérieu 2a and 2c (Ain, France) and the MN11 sites of Ambérieu 1 (Ain, France) and Bernardière (Drôme, France) [34], [35]. In Spain, this taxon has been recovered from the late MN10 localities of Can Perellada, Santa Margarita, and Can Jofresa [36], Cal Turu, Creu Conill 10, Ceramiques Viladecavalls, Torrent de Febulines 3, M, Trinxera Sud Autopista 1, 2, 3, Trinxera Nord Autopista and Trinxera Nord Autopista 2, and Viladecavalls Km 7 (Barcelona) [23], [24], [37]. In addition, the presence of *R*. cf. *bressanus* has been mentioned [35] for the late MN10 site of Dionay (Isère, France).

The cheek teeth of *Rotundomys bressanus* are larger, more lophodont, and have deeper valleys than those of *R. intimus* sp. nov. The upper molars of *R. bressanus* have the posteroloph completely fused with the metalophule, whereas there are various specimens in which the posteroloph exceeds its junction with the metalophule in the sample from Batallones. Besides, most of the M1 of *R. bressanus* have a spur of the anterolophule, which connects the protocone to the labial anterocone, whereas most of the M1 of *R. intimus* sp. nov. lack it. The M2 of *R. bressanus* show a strong mesocone and usually a short but distinct mesoloph, which are absent on most of the equivalent teeth from Batallones. In addition, the m1 of *R. bressanus* have a well-marked and high anterolophulid and a well-developed metalophulid, whereas the anterolophulid and metalophulid are low, weak, and interrupted (or even absent) in most of the m1 of *R. intimus* sp. nov. Moreover, *R. bressanus* is characterized by the reduction of the labial anterolophid on the m1, which is present in *R. intimus* sp. nov.

#### Comparison with *Rotundomys mundi* Calvo, Elizaga, López-Martínez, Robles et Usera, 1978 [16]

This species was created on the basis of some isolated cheek teeth from the MN10 locality of Hijar-1 (Albacete, Spain). The holotype (H-7) is a right M2. We were unable to locate any of the specimens mentioned in [16] despite our efforts, so the present whereabouts should be considered as unknown. Agustí ([22]: 136) has described *Rotundomys* cf. *mundi* from the late MN10 localities of Trinxera Nord Autopista, Trinxera Nord Autopista II, Trinxera Sud Autopista, and Can Perellada (Barcelona, Spain). Later [36] he changed this assignation into *Rotundomys* sp.. However, according to Freudenthal et al. [7] this material would in fact correspond to *R. mundi*, the first interpretation of Agustí [22] being accurate.

*R. mundi* is based on a very small number of specimens. As far as we can judge of it, *R. sabatieri*, which is known from many more specimens, is not fundamentally different from it. However, the scoring of these two species differs (e.g., characters 29 and 34) so that we provisionally accept them as distinct pending further investigations.

The morphology of the single recorded M2 of *Rotundomys mundi* is very different from that of the equivalent teeth of *R. intimus* sp. nov. The former has a complete anterior metalophule that is absent on the M2 of *Rotundomys* from Batallones, in which the metalophule is short and posterior. In addition, the M3 of *R. mundi* are much more reduced than those of *R. intimus* sp. nov. and they have a strong connection between the paracone and the labial anteroloph, which is unknown on the M3 of *R. intimus* sp. nov.

#### Comparison with *Rotundomys montisrotundi* (Schaub, 1944) [14]

This species was originally erected as *Cricetodon montisrotundi* by Schaub [14]. Subsequently, Mein [13] created the new genus *Rotundomys* and reallocated this species to it. The holotype, an isolated left m1, comes from the Late Miocene (MN10) site of Montredon (Hérault, France). It is housed in NMB.

Additional material of this species has been uncovered from French and Spanish MN10 localities. In France, this taxon has been found in Lo Fournas 7, Lo Fournas 6, Pyrénées-orientales [38], Lo Fournas 1993 [35], Les Bourbons, Drôme [39] and, in Spain, in Can Casablanques 2, Barcelona [40], Ampudia 3, Palencia [27], [28], [29], and from the MN10-MN11 site of Racor, Almería [41]. Furthermore, *Rotundomys* cf. *montisrotundi* is cited from the MN10 locality of Douvre (Ain, France) [35] as well as from the localities of Can Llobateres (MN9b/MN10) and Can Casablanques (MN10) (Barcelona, Spain) on the basis of scarce material ([42], [22]: 117). According to Casanovas-Vilar ([24]: 77), the single molar of *Rotundomys* from Can Llobateres 1 assigned by Agustí [22] to *R.* cf. *montisrotundi* would be in fact *Rotundomys* sp.

On the basis of the rich sample of *Rotundomys montisrotundi* from Montredon (Herault, France), Aguilar [33] described various morphotypes that he found on the cheek teeth of this species. We found some of these morphotypes on the check teeth of *R. intimus* sp. nov. but in different percentages in the Spanish and French populations. For instance, 28% of the m1 show the morphotype i (specimens with the metaconid isolated and a longitudinal anterolophulid connected to the anterolophid) in Batallones 5, whereas it has been found only in 9% of the specimens from Montredon. The connections between the metalophulid and the anterolophid or between the anterolophulid and the anterolophid are weaker in the Batallones sample than in *R. montisrotundi*. Moreover, some m1 of *R. intimus* sp. nov. have a weak but distinct posterior metalophulid, which is absent in *R. montisrotundi*. On the whole, the molars of *R. intimus* sp. nov. are less lophodont than those *R. montisrotundi*. In fact, in the former species all cusps/ids are very distinct. In particular, the metacone is large and the cusp/ids of the anteroloph and anterolophid are distinct in the first molars. The cingula of *R. intimus* sp. nov. are less strong and the valleys shallower than in *R. montisrotundi*. *R. intimus* sp. nov. is more robust than *R. montisrotundi*.

#### Comparison with *Rotundomys sabatieri* Aguilar, Michaux et Lazzari, 2007 [18]

This species has been erected on the basis of numerous isolated cheek teeth from the Turolien locality of Lo Fournas 16-M (Pyrénées-Orientales, France). Its holotype (Fou 16-M n°395) is a right m1 that is housed in USTL. Further material of this taxon has been recovered from Lo Fournas 6 (Pyrénées-Orientales, France) [18].

The cheek teeth of *Rotundomys sabatieri* are more lophodont with less distinct cusps and are less robust than those of *R. intimus* sp. nov. Moreover, all M1 of *R. sabatieri* have the metalophule completely fused with the posteroloph and lack the labial posterosinus (morphotype c according to Aguilar [33]). On the contrary, on the M1 of *R. intimus* sp. nov. the metalophule is not completely fused with the posteroloph, leaving a small labial posterosinus, which disappears with wear. In addition, some of the M1 of *R. sabatieri* show a short but distinct mesoloph (or incomplete anterior metalophule) directed towards the metacone, whereas a true mesoloph is never present on the M1 of *R. intimus* sp. nov. (instead, there is a thickening of the anterior arm of the hypocone). Some of the M2 of *R. sabatieri* show a double metalophule (morphotypes d and e according to Aguilar [33]), whereas none of *R. intimus* sp. nov. show it. With respect to the lower molars, the m1 of *R. sabatieri* have strong connections of metalophulid-anterolophid and anterolophulid-anterolophid, which are weak, interrupted or even absent on the equivalent teeth of the Batallones sample.

#### Comparison with *Rotundomys freiriensis* Antunes et Mein, 1979 [17]

This species was coined on the basis of 21 teeth recovered from the lower MN10 site of Freiria do Rio Maior (Santarém, Portugal) [17]. Its holotype, an isolated left m1, is housed at the Stratigraphical and Palaeobiological center of UNL. Additional material of this species has not been found to date.

*Rotundomys freiriensis* is smaller than *R. intimus* sp. nov. The m1 of *R. freiriensis* lack the anterolophulid and have a transverse metalophulid connected to the protoconid instead of the anteroconid. All m1 of *R. intimus* sp. nov. have a distinct anterolophulid and the metalophulid points always strongly forwards, nearly longitudinal. Furthermore, the m2 of *R. freiriensis* have a distinct lingual anterolophid that is absent on the m2 of *R. intimus* sp. nov. The M3 of *R. freiriensis* are much more reduced than those belonging to the Batallones sample.

## Discussion

The general morphological pattern of *Rotundomys intimus* sp. nov. recalls that of *R. montisrotundi* and *R. sabatieri*. However, the detailed comparison described above between the type material of the latter species and *R. intimus* sp. nov. reveals the existence of important differences between these taxa that justify the erection of the new species. *R. intimus* sp. nov. is characterized by being less lophodont, having higher cusps/ids, weaker connections, shallower valleys, and thinner cingula than *R. montisrotundi* and *R. sabatieri*. There are also size differences between the type material of these taxa and the samples from Batallones.

**Figure 8:**
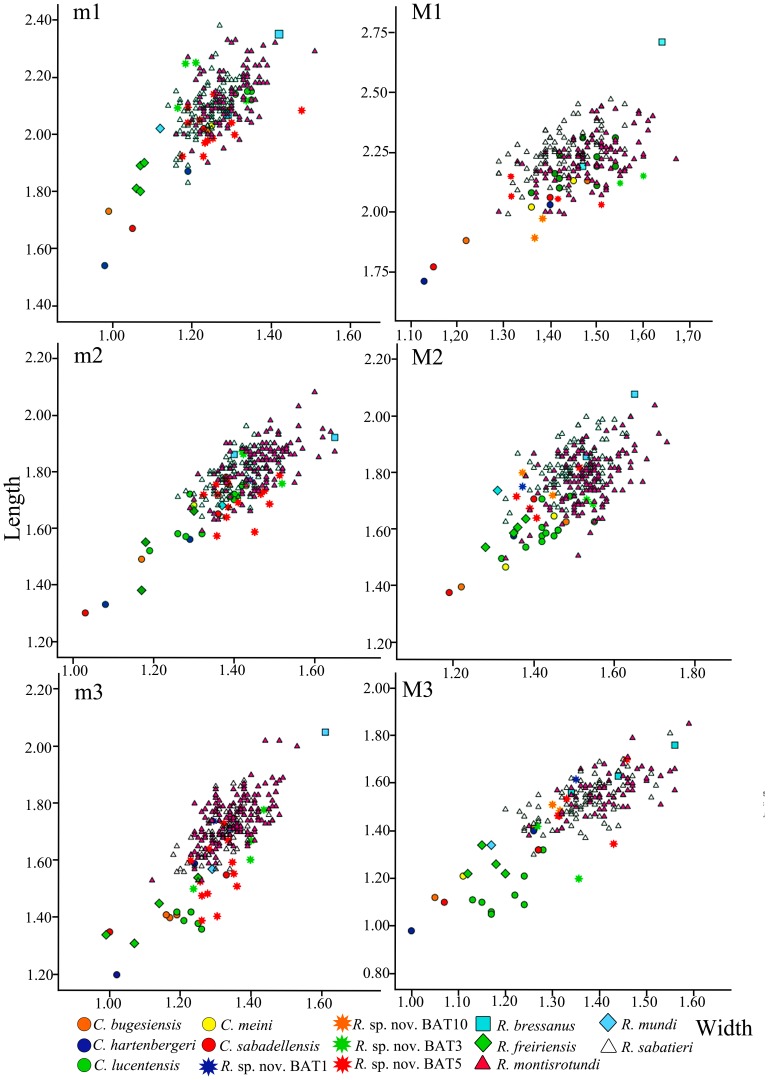
Length/width scatter diagrams of the upper and lower molars of the species belonging to the genera *Cricetulodon* and *Rotundomys*.

Figure 8 shows the scatter plot of the maximum length and width of the dental elements of all species belonging to the genus *Rotundomys*. It clearly shows the differences in size between *R. intimus* sp. nov. and the remaining species of the genus. The specimens from Batallones 5 are distributed in the lower range of *R. montisrotundi*, *R. bressanus* and *R. sabatieri* and in the upper range of *R. freiriensis* and *R. mundi*.

Several variance analyses (ANOVA) have been performed for the length and width of each dental element to appreciate the differences amongst the samples (Text S3). The results show significant differences for most dental elements between the population from Batallones 5 and *Rotundomys montisrotundi* on one hand and *R. sabatieri* on the other hand. In fact, with the exception of the M3, the width of the m1 and the length of the M2, the Tukey's Honest Significant Differences post-hoc test indicates that the length and width of the molars of *R. montisrotundi* are significantly larger than those of *R. intimus* sp. nov. from Batallones 5. The differences in size that we have found in these samples are particularly meaningful taking into consideration that the population of Montredon presents a very wide range of size dispersion (see Figure 8).

With regard to *R. sabatieri*, this test shows that the length of the M1, m2, and m3 are significantly larger than in *R. intimus* sp. nov. from Batallones 5.

### Phylogeny

In order to elucidate the relationships between the species pertaining to the genera *Cricetulodon* and *Rotundomys* and the position of the new species from Batallones within *Rotundomys*, the first cladistic analysis involving all species of these genera has been conducted.

**Figure 9:**
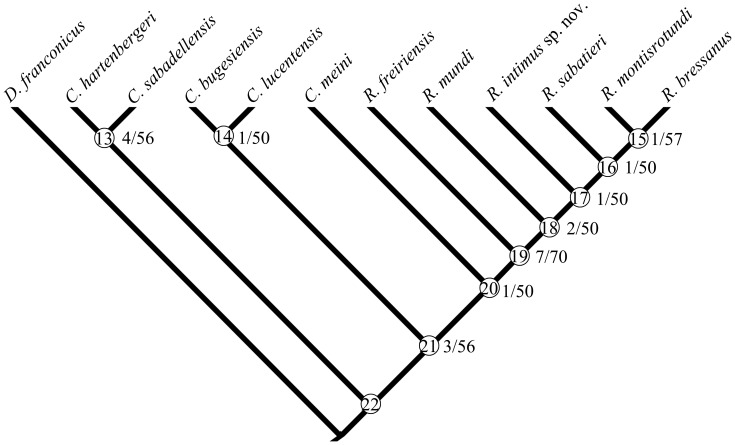
Single most parsimonious tree generated by the cladistic analysis of *Cricetulodon* and *Rotundomys* performed in this paper (matrix in Appendix 1). Nodes are circled and designed by numbers 22-13; Bremer and relative Bremer indices are shown at the appropriate nodes.

A single most parsimonious tree has been generated with a length of 67 and a low degree of homoplasy (CI = 0.746 and RI = 0.825). Branch support was estimated through two complementary indices: Bremer Support [9] and Relative Bremer Support [10]. These indices are indicated for each node on the cladogram in the figure 9. It should be stressed that some of them are as low as 1, including that from which *Rotundomys intimus* arises. On a side note, no difference in topology (only very slight CI and RI deviations) occurs when serial homologues (characters 14, 16, 17, 18, and 37) are run as single characters.

The tree shows a completely resolved topology. *Cricetulodon hartenbergeri* and *C. sabadellensis* position as sister-species in the most basal branch. *C. bugesiensis* and *C. lucentensis* split as sister-species at the base of a clade that is one node less inclusive. *C. meini* inserts between them and the remaining species of the ingroup, which all belong to *Rotundomys*. These species are henceforth fully asymmetrically distributed along the crown of the cladogram in an arrangement that lines up from *R. freiriensis* to *R. bressanus* plus *R. montisrotundi*. *R. intimus* sp. nov. locates in the middle of this sequence, in which it is flanked basally by *R. mundi* and apically by *R. sabateri*.

However as explained above (§ comparison), the available material of *R. mundi* is very scarce so far so that only about 60% of the characters could be scored. Thus, this taxon is prone to shift its phylogenetical position, given new information. However, if we prune *Rotundomys mundi* from the ingroup before running the analysis the topology of the tree obtained is not altered, which means that this species is not currently affecting the results of our analysis.

The transformations supporting the topology of this tree (under the ACCTRAN and DELTRAN optimizations) are listed in Table 3. Each internal node is discussed below, beginning from the most basal (whenever both unambiguous and ambiguous synapomorphies support a given node, only the former are mentioned).

**Table 3:** Synapomorphies plotted in the most parsimonious tree under ACCTRAN and DELTRAN optimizations.

	Acctran	Deltran
*Democricetodon*→Node 22	**19(0→1); 31(0→1); 35(0→1)**	**19(0→1); 31(0→1); 35(0→1)**
Node 22→Node 13	8(2→1)**; 16(2→0);** *22(1→0)* **; 40(1→0); 41(1→0)**	8(2→1); **16(2→0); 40(1→0); 41(1→0)**
Node 13→*C. sabadellensis*	3(0→1); 26(0→1)	3(0→1); 26(0→1)
Node 12→*C. hartenbergeri*		*22(1→0)*
Node 22→ Node 21	**3(0→2); 9(0→2); 11(0→1); ** ***18(0→1)***	**3(0→2); 9(0→2); 11(0→1)**
Node 21→ Node 14	**8(2→0)**; 5(0→1); 36(0→1)	**8(2→0)**; 5(0→1);36(0→1)
Node 14→*C. bugesiensis*	3(2→1); 15(0→1); *18(1→0)*;33(0→1); 34(0→1)	3(2→1); 15(0→1); 33(0→1); 34(0→1)
Node 14→*C. lucentensis*	10(0→1); 17(0→1); 19(1→0); 20(0→1); 23(0→1)	10(0→1); 17(0→1); *18(0→1)*; **19(1→0)**; 20(0→1); 23(0→1)
Node 21→ Node 20	***7(0→1)*** **; 15(0→2)** ***;38(0→1)*** **; ** ***39(0→1)*** **; ** ***42(0→1)***	**15(0→2)**; *18(0→1)*
Node 20→*C. meini*	20(0→1); **24(2→0)**	20(0→1); **24(2→0)**
Node 20→ Node 19	*1(0→1)*; **6(0→2)**; *10(0→1)*; **13(0→1)**; 14(0→1); **21(0→1); ** ***25(0→1)***; 26(0→1); **27(0→1)**; 30(0→1); ***31(1→2)*** *; * ***32(0→1)***	**6(0→2); ** ***7(0→1)*** **; 13(0→1)**; 14(0→1); **21(0→1)**; 26(0→1); **27(0→1)**; 30(0→1); ***38(0→1)*** **; ** ***39(0→1***
Node 19→ Node 18	***1(1→2)***; *5(0→1)*; *8(2→1)*; **28(0→2)**; 33(0→1); 36(0→1)	***1(0→2)*** **; 28(0→2); ** ***31(1→2)***; 33(0→1); 36(0→1)
Node 18→ Node 17	**29(0→1)**; 34(0→1)	*5(0→1); 8(2→1)*; **29(0→1)**; 34(0→1)
Node 17→ Node 16	**2(0→1); 4(0→1)**; 15(2→1)	**2(0→1); 4(0→1)**; 15(2→1)
Node 16→ Node 15	**12(0→1)**; *17 (0→1)*; ***37(0→1)***	**12(0→1)**
Node 15→*R. montisrotundi*		
Node 15→*R. bressanus*	**27(1→2)**	*10(0→1); 17(0→1)*; ***25(0→1)*** **; 27(1→2); ** ***32(0→1); 37(0→1)*** **; ** ***42(0→1)***
Node 17→*R. intimus* sp. nov.	*1(2→1)*; **28(2→1)**	*1(2→1)*; **28(2→1)**
Node 18→*R. mundi*	15(2→0)	15(2→0)
Node 19→*R. freiriensis*	3(2→1); 23(0→1); ***31(2→3)***	*1(0→1)*; 3(2→1); 23(0→1); ***31(1→3)***

Exclusive synapomorphies are indicated in bold. Italics indicate ambiguous synapomorphies. Node numbers are shown in Figure 9.

Node 22 (Ingroup). This clade is supported by three exclusive and unambiguous synapomorphies: LM1/LM3 ratio between 1.78 and 1.58; anterolophulid mostly joined with the lingual cusp of the anterolophid (this character is lost at node 19, all taxa arising from this node have the anterolophid mostly connected to the labial cusp of the anterolophid except for *R. freiriensis*, which lacks the anterolophulid); absence of mesolophid on the m2.

Node 13 (*Cricetulodon hartenbergeri* + *Cricetulodon sabadellensis*). Three exclusive and unambiguous synapomorphies support this clade: M2 with nearly transverse posterior metalophule, presence of mesolophid and metalophulid connected to the anterolophulid behind the anteroconid on the m3. This node is supported by an additional unambiguous and non-exclusive synapomorphy: the frequent absence of anterior metalophule on the M1 (a parallelism with node 18 under ACCTRAN and node 17 under DELTRAN).

Node 14 (*Cricetulodon bugesiensis* + *Cricetulodon lucentensis*). This clade is supported by an unambiguous and exclusive synapomorphy: absence of anterior metalophule on the M1. Two non-exclusive synapomorphies are also present at this node: presence of a forked anterolophule in some M1 (a parallelism with node 18 under ACCTRAN and with node 17 under DELTRAN); metalophulid connected to the anteroconid on the m2 (a parallelism with node 18).

Node 21 (*Cricetulodon bugesiensis* + *Cricetulodon lucentensis*) + more derived species. Three unambiguous and exclusive synapomorphies support this node: m1 longer than 1.95 mm (this character is lost in *Cricetulodon bugesiensis* and *Rotundomys freiriensis*, which have a length of the m1 between 1.70 and 1.95 mm), M1 with posterior metalophule fused with the posteroloph or very oblique backwards, and absence of labial posterosinus.

Node 20 (*Cricetulodon meini* + more derived). A single unambiguous and exclusive synapomorphy supports this node: absence of anterior metalophule on the M2 (this character is reversed in *Rotundomys mundi*, in which this structure is present in some specimens, and in the taxa arising from node 16).

Node 19 (*Rotundomys freiriensis* + more derived species). The clade *Rotundomys* is sustained by four exclusive and unambiguous synapomorphies: loss of the anterior protolophule on the M1-M3, protoconid on the m1 connected to the hypolophulid in a regularly curved crest. In addition, three non-exclusive and unambiguous synapomorphies support this node as the absences of: mesoloph on the M2 (a parallelism with some specimens belonging to *Cricetulodon sabadellensis*, *C. lucentensis*, and *C. bugesiensis*), labial posterosinus on the M3 (a parallelism with *C. sabadellensis*) and mesolophid on the m1 (a parallelism with some specimens of *C. sabadellensis*, and *C. bugesiensis*). The clade *Rotundomys* roots at this node. In ACCTRAN, a synapomorphic moderate lophodonty is assumed to occur here and then to be further pronounced at the following node without any reversion in more derived species but *Rotundomys intimus*, which has distinct cusps.

Node 18 (*Rotundomys mundi* + more derived species). Two unambiguous and exclusive synapomorphies support this node: increase in lophodonty (see above) and presence of a nearly longitudinal metalophulid on the m1. The metalophulid is strong except for *Rotundomys intimus* sp. nov., in which it is weak. Two unambiguous non-exclusive synapomorphies support this clade: absence of anterosinusid on the m2 (a parallelism with *C. bugesiensis*) and metalophulid connected to the anteroconid on the m2 (a parallelism with the taxa of Node 14).

Node 17 (*Rotundomys intimus* sp. nov.+ more derived species): This node is supported by the unambiguous and exclusive synapomorphy of having some m1 with the metalophulid and anterolophulid connected by a transverse ridge and the unambiguous and non-exclusive synapomorphy of lacking lingual anterolophid on the m2 (a parallelism with *Cricetulodon bugesiensis*).

Node 16 (*Rotundomys sabatieri* + more derived species). Two exclusive and unambiguous synapomorphies support this node: deep valleys and a crest-like anteroconid. In addition, an unambiguous non-exclusive synapomorphy sustains this clade: anterior metalophule on the M2 mostly absent (a parallelism with *Cricetulodon bugesiensis*).

Node 15 (*Rotundomys bressanus* + *Rotundomys montisrotundi*). This clade is supported by a single unambigous and exclusive synapomorphy: the absence of labial anteroloph on most of the M2.

The relationships between and within the genera *Cricetulodon* and *Rotundomys* have been dealt with in only a few articles. According to Freudenthal ([19]: fig. 4), *Cricetulodon hartenbergeri* is the ancestor of *C. sabadellensis*, which is itself the predecessor of *R. montisrotundi* in the same lineage. This derivation would be marked by an increase in hypsodonty and size and a reduction of the mesolophs, mesolophids, and anterior protolophules ([19]: 313). However, Agustí [43] advocated that this hypothesis could not be maintained because of the coexistence of *C. sabadellensis* and *R. montisrotundi* in Can Llobateres. Freudenthal et al. [7] agreed with this opinion, provided that this coexistence is real, and suggested in this case two lineages for these species, whose dichotomy would have taken place shortly prior to the formation of the Can Llobateres site. Regardless of the fact that the co-occurrence of two species in a given site does not preclude that one could be the descendant of the other, the presence of *R. montisrotundi* in Can Llobateres is questioned [24].

Mein [13] suggested the following evolutionary sequence: *Cricetulodon hartenbergeri*-*C. sabadellensis*-*R. montisrotundi*-*R. bressanus*. Freudenthal et al. [7] mentioned that he could not find any argument against the derivation of *R. bressanus* from *R. montisrotundi*. The *R. montisrotundi*-*R. bressanus* lineage is certainly not in opposition with the topology of our cladogram.

Antunes and Mein [17] described a new species, *Rotundomys freiriensis*, considered as more derived than *Cricetulodon sabadellensis* and C. *hartenbergeri* by the loss of mesolophs and mesolophids, anterior protolophules and posterosinus, and the anterolingual cingulum on the m3. They derived *C. sabadellensis* from *C. hartenbergeri* ([17]: tab. 1), and from *C. sabadellensis*, two lineages would have evolved: *R. freiriensis*-*R. mundi* and *R. montisrotundi*-*R. bressanus* ([17]: tab. 1). According to our results, *C. sabadellensis* and *C. hartenbergeri* are sister-species. They are separated from *R. freiriensis* by a number of species (*C. bugesiensis*, *C. lucentensis*, and *C. meini*). *R. freiriensis* is indeed, as advocated by Antunes and Mein [17], a more primitive taxon than *R. montisrotundi*, *R. bressanus*, and *R. mundi*. However, they are all derivatives of one stem: the existence of two lineages starting with *R. freiriensis* and *R. montisrotundi*, respectively, is not supported by the results of our analysis.

According to Aguilar [33], there would be a single lineage of *Rotundomys* in which the small and morphologically simple *R. freiriensis* is a forerunner of *R. montisrotundi* (of which *R. bressanus* and *R. mundi* would be junior synonyms). This schematic view of the origin and evolution of *Rotundomys* is not in contradiction with our topology. Aguilar [33] considered *R*. *freiriensis* as an off-shoot of the *Cricetulodon* lineage that had developed from *C. sabadellensis*.

Freudenthal et al. [7] re-iterated that *Cricetulodon hartenbergeri* may well have given rise to *C*. *sabadellensis* through moderate size increase, the development of trilobate anteroconids, the reduction of the mesolophs, mesolophids, and anterior protolophule. In addition, *C. hartenbergeri* supposedly gave rise independently to *C. bugesiensis* (through an enlargement and a simplification of the dental pattern) and *C. lucentensis*, the latter through *C. meini.* Freudenthal et al. [7] considered that *C*. *sabadellensis* shows an advanced morphology that is consistent with it being the ancestor of a species of *Rotundomys* like *R. montisrotundi* through increased hypsodonty, the developing of a flat wear surface with equally high crests and cusps, the loss of the mesolophid in m1 and m2, the loss of the anterosinusid in m2, the reduction of the anterior protolophule, and of the mesoloph. Freudenthal et al.'s [7] conjectures about the evolution of the species of *Cricetulodon* are not supported by the results of our analysis, which pose (*C. hartenbergeri*, *C. sabadellensis*), (*C. bugesiensis*, *C. lucentensis*), and *C. meini* as successively closer outgroups to *Rotundomys*, whose basalmost species is *R. freiriensis*.

More recently, Kälin ([44]: fig. 36.4) and Fejfar et al. ([4]: 16) advocated the existence of a lineage originating from *Cricetulodon sabadellensis* and arriving at *R. montisrotundi* through *C. hartenbergeri.* This is at odds with the opinion of Freudenthal et al. [7] and other authors who suggested that it is *C. hartenbergeri* that could have given rise to *C. sabadellensis* and not the other way around. Our results do not lend credence to one view over the other.

Our results suggest that *Cricetulodon* represents a paraphyletic assemblage of species basal to *Rotundomys* spp. They do not, however, indicate whether an alternative topology with a monophyletic *Cricetulodon* would be statistically rejected. If the species of *Cricetulodon* are constrained to form a monophyletic (unresolved) assemblage with sister-group relationships with the clade *Rotundomys*, then the tree is 80 steps long. A templeton test as implemented in PAUP* version 4.0b10 (Sinauer Associates, Sunderland, MA, USA) using the PaupUp graphical interface (Calendini F & Martin JF, Montpellier, France) was used to evaluate this topology with respect to that of the 67-step most parsimonious tree. It revealed that these two topologies are significantly different: in other words, our character/taxon matrix is confidently more compatible with the optimal tree than with the constrained one.

*Cricetulodon* can be seen as a “basal stock” from which the first species of *Rotundomys* was eventually derived. As for the taxonomy, our topology suggests that only *Cricetulodon sabadellensis* and *C. hartenbergeri* can be retained in this genus. The species *C. bugesiensis*, *C. lucentensis*, and *C. meini* would be best considered as actually pertaining to *Rotundomys*. However, pending further and more comprehensive phylogenetic analyses we refrain from formally reallocating these three species at this stage. We, therefore, continue using *Rotundomys* in the narrower sense used by Freudenthal et al. [7] and others.

The evolution of *Rotundomys* is marked by the development of lophodonty on the cheek teeth, the loss of the anterior protolophule on the upper molars, the connection between protoconid and hypolophulid in a regularly curved crest, and the complete loss of mesolophs and mesolophids. However, the absence of the mesoloph on the M1 and M2 and the mesolophid on the m1 is also found in some specimens of several species of *Cricetulodon*. This suggests that these characters were not stable in populations of *Cricetulodon*, but quickly became so in the course of *Rotundomys* evolution. The mesolophs and the mesolophids are lost on the M3, m2, and m3 earlier than on the remaining teeth. The evolution from a nearly transverse to a very much oblique backwards posterior metalophule, commonly fused with the posteroloph on the upper molars, begins before the establishment of the *Rotundomys* clade. The same holds true for the loss of the anterior metalophule on the M1 and M2, which occurs in most specimens of *Rotundomys* spp. Nevertheless, this structure is also lost in most or all M1 of some species of *Cricetulodon* (C. *sabadellensis*, *C. hartenbergeri*, and *C. meini*) and on the M2 of *C. bugesiensis* and *C. meini*.

*Rotundomys intimus* sp. nov. shares one exclusive and unambiguous synapomorphy with the more derived species of the genus: an occasional transverse connection between the metalophulid and the anterolophulid on the m1 (character 29, state 1). These species also present the unambiguous synapomorphy of having lost the lingual anterolophid on the m2 (character 34, state 1), which is not exclusive as it is also found homoplastically (parallelism) in *Cricetulodon bugesiensis*. Two other possible synapomophies are the anterolophule that is forked in part of the M1 at least (character 5, state 1) and the anterior metalophule that is absent in most, but not all, of the M1 (character 8, state 1). They are, however, equivocal (the M1 is unknown in *R. mundi*, which flanks basally *R. intimus* sp. nov.) and, in any event, non-exclusive as the former character-state has been acquired independently in (*C. bugesiensis* +*C. lucentensis*) and the latter in (*C. hartenbergeri* +*C. sabadellensis*). On the other hand, *R. intimus* sp. nov. is drawn aside from the clade formed by the most evolved species of *Rotundomys* by its archaic lophodonty showing distinct cusps (character 1, state 1), the shallow depth of the valleys of the occlusal surface (character 2, state 0), and the subdivided anteroconid on the M1 (character 4, state 0). Indeed, in *R. sabatieri, R. montisrotundi*, and *R. bressanus* the lophodonty is perfected, the valleys are deep, and the anteroconid on the M1 is crest-shaped (all are exclusive and unambiguous synapomorphies). In addition, in these species the anterior metalophule is mostly absent on the M2 (unambiguous synapomorphy), as it occurs homoplastically in *C. bugesiensis*, whereas it is always absent in *R. intimus* sp. nov. (a character-state acquired in the ancestor of *C. meini* and more derived species, but reversed in *R. mundi*). The moderately developed lophodonty of *R. intimus* sp. nov. can be optimised as either a reversion to the state that appears at node 19 (ACCTRAN) or as a parallelism with *R*. *freiriensis* (DELTRAN). However, the hypothesis of a reversion is not very plausible because once the lophodonty is acquired within a lineage, it is retained. The fact that the phylogenetical position of *Rotundomys mundi* is uncertain due to the amount of missing data makes it possible for *Rotundomys intimus* to be actually the second most basal taxon inside *Rotundomys*, i.e. not as distant from *R. freiriensis* as reflected in our analysis.

## Supporting Information

Text S1 List of Characters and character states used for the phylogenetic analysis.
(DOCX)

Text S2 Character/taxon matrix used in the analysis of relationships of all species of *Cricetulodon* and *Rotundomys*. Characters are listed in Text S1. Character scoring: 0, 1 and 2, conditions of character;?, character state uncertain.
(DOCX)

Text S3 Analyses of the variance (ANOVA), including the Levene test for Homogeneity of Variances, for Length (L) and Width (W) of the Type material of *Rotundomys freiriensis* from Freiria do rio Maior, *R. intimus* nov. sp. from Batallones 5, *R. sabatieri* from Lo Fournas 16M, and *R. mantisrotundi* from Montredon. The last four columns indicate the homogeneous subsets calculated by Tukey's Post Hoc test (alpha  =  0.05).
(DOCX)
