# Supplementary material for: New Species of Rotundomys (Cricetinae) from the Late Miocene of Spain and Its Bearing on the Phylogeny of Cricetulodon and Rotundomys
Source: PLoS One. 2014 Nov 12;9(11):e112704. doi: 10.1371/journal.pone.0112704 (PMC4229238; doi:10.1371/journal.pone.0112704)
Supplement: Text S1 — List of Characters and character states used for the phylogenetic analysis. (DOCX) [file pone.0112704.s001.docx]

**TEXT S1 List of Characters and character states used for the phylogenetic analysis**

1. (0) Bunodont; (1) Lophodont but with distinct cusps; (2) Lophodont.
2. Depth of valleys (0) shallower than *R. montisrotundi*; (1) as deep as or deeper than *R. montisrotundi*.
3. Size: (0) Lm1≤ 1.7; (1) 1.7<Lm1≤1.95; (2) Lm1>1.95.

**M1:**

1. Anterocone: (0) subdivided; (1) crest-like.
2. Anterolophule: (0) simple; (1) forked at least in some specimens.
3. Protolophule anterior: (0) present; (1) incomplete; (2) absent.
4. Mesoloph: (0) present; (1) absent.
5. Anterior metalophule:(0) mainly present; (1) mainly absent; (2) absent.
6. Posterior metalophule: (0) oblique backwards; (1) very much oblique backwards; (2) absent (posteroloph is fused with the metalophule).
7. Labial cingula: (0) present; (1) absent.
8. Labial posterosinus: (0) present; (1) absent.

**M2:**

1. Labial anteroloph: (0) present; (1) absent at least in some specimens.
2. Anterior protolophule: (0) present; (1) absent.
3. Mesoloph: (0) present; (1) absent.
4. Anterior metalophule: (0) mostly present; (1) mostly absent; (2) absent.
5. Posterior metalophule: (0) oblique backwards (but nearly transverse), (1) very much oblique backwards; (2) absent (fused with the posteroloph).
6. Labial cingula: (0) present; (1) absent.
7. Labial posterosinus: (0) present; (1) absent.

**M3:**

1. LM1/LM3: (0)1.98-1.78; (1)1.78-1.58.
2. Lingual anteroloph: (0).present; (1).absent.
3. Anterior protolophule: (0) present; (1) absent.
4. Mesoloph: (0) present; (1) absent.
5. Anterior metalophule: (0) present; (1) absent at least in some specimens.
6. Posterior metalophule: (0) slightly oblique; (1) very much backwards (2) absent (posteroloph is fused with the metalophule).
7. Labial cingula: (0) present; (1) absent.
8. Labial posterosinus: (0) present; (1) absent.

**m1**

1. Protoconid: (0) connected to the hypolophulid but not in a regularly curved crest; (1) connected to the hypolophulid in a regularly curved crest; (2) connected to the hypolophulid in a bent crest.
2. Metalophulid: (0) oblique fordwards (nearly transverse); (1) weak and strongly forwards (nearly longitudinal); (2) strong and nearly longitudinal.
3. Transverse connection between metalophulid and anterolophulid: (0) absent; (1) may be present.
4. Mesolophid: (0) present; (1) absent.
5. Anterolophulid: (0) central; (1) mostly joined with the lingual cusp of the anterolophid; (2) mostly connected to the labial cusp of the anterolophid; (3) absent.
6. Lingual cingula: (0) present; (1) absent.

**m2**

1. Anterosinusid: (0) present; (1) absent.
2. Lingual anterolophid: (0) present; (1) absent.
3. Mesolophid: (0) present; (1) absent.
4. Metalophulid: (0) connected to the anterolophulid, behind the anteroconid; (1) connected to the anteroconid.
5. Lingual cingula: (0) present; (1) absent.

**m3**

1. Anterosinusid: (0) present; (1) absent.
2. Lingual anterolophid: (0) present; (1) absent.
3. Mesolophid: (0) present at least in some specimens; (1) absent.
4. Metalophulid (0) connected to the anterolophulid, behind the anteroconid; (1) connected to the anteroconid.
5. Lingual cingulum: (0) present; (1) absent.
