# Supplementary material for: New Species of Rotundomys (Cricetinae) from the Late Miocene of Spain and Its Bearing on the Phylogeny of Cricetulodon and Rotundomys
Source: PLoS One. 2014 Nov 12;9(11):e112704. doi: 10.1371/journal.pone.0112704 (PMC4229238; doi:10.1371/journal.pone.0112704)
Supplement: Text S2 — Character/taxon matrix used in the analysis of relationships of all species of Cricetulodon and Rotundomys. Characters are listed in Text S1. Character scoring: 0, 1 and 2, conditions of character;?, character state uncertain. (DOCX) [file pone.0112704.s002.docx]

|  | 1 | 2 | 3 | 4 | 5 | 6 | 7 | 8 | 9 | 10 | 11 | 12 | 13 | 14 | 15 | 16 | 17 | 18 | 19 | 20 | 21 |
| --- | --- | --- | --- | --- | --- | --- | --- | --- | --- | --- | --- | --- | --- | --- | --- | --- | --- | --- | --- | --- | --- |
| *Democricetodon franconicus* | 0 | 0 | 0 | 0 | 0 | 0&1 | 0 | 2 | 0 | 0 | 0 | 0 | 0 | 0 | 0 | 2 | 0 | 0 | 0 | 0 | 0 |
| *Cricetulodon hartenbergeri* | 0 | 0 | 0 | 0 | 0 | 0 | 0 | 1 | 0 | 0 | 0 | 0 | 0 | 0 | 0 | 0 | 0 | 0 | 1 | 0 | 0 |
| *Cricetulodon sabadellensis* | 0 | 0 | 1 | 0 | 0 | 0&1 | 0 | 1 | 0 | 0 | 0 | 0 | 0 | 0&1 | 0 | 0 | 0 | 0 | 1 | 0 | 0 |
| *Cricetulodon bugesiensis* | 0 | 0 | 1 | 0 | 1 | 0&1 | 0 | 0 | 1&2 | 0 | 0&1 | 0 | 0 | 0&1 | 1 | 0&2 | 0 | 0 | 0 | 0 | 0 |
| *Cricetulodon meini* | 0 | 0 | 2 | 0 | 0 | 0 | 0&1 | 2 | 2 | 0 | 1 | 0 | 0 | 0 | 2 | 2 | 0 | 1 | 0 | 1 | 0 |
| *Cricetulodon lucentensis* | 0 | 0 | 2 | 0 | 1 | 0&1 | 0&1 | 0 | 1&2 | 1 | 1 | 0 | 0 | 0&1 | 0 | 1&2 | 1 | 1 | 0 | 1 | 0 |
| *Rotundomys montisrotundi* | 2 | 1 | 2 | 0&1 | 1 | 1&2 | 1 | 1 | 1&2 | 0&1 | 0&1 | 1 | 1 | 1 | 1 | 1&2 | 0&1 | 0&1 | 1 | 0 | 1 |
| *Rotundomys bressanus* | 2 | 1 | 2 | 1 | 1 | 1&2 | 1 | 1 | 2 | 1 | 1 | 1 | 1 | 1 | 1 | 2 | 1 | 1 | 1 | 0 | 1 |
| *Rotundomys mundi* | 2 | ¿ | 2 | ¿ | ¿ | ¿ | ¿ | ¿ | ¿ | ¿ | ¿ | 0 | 1 | 1 | 0 | 2 | 0 | 1 | ? | 0&1 | 1 |
| *Rotundomys sabatieri* | 2 | 1 | 2 | 1 | 1 | 2 | 1 | 1 | 2 | 0&1 | 1 | 0 | 1 | 1 | 0&1 | 2 | 0 | 1 | 1 | 0 | 1 |
| *Rotundomys freiriensis* | 1 | 0 | 1 | 0 | 0 | 2 | 1 | 2 | 2 | ¿ | 1 | 0 | 1 | 1 | 2 | 2 | ¿ | 1 | 1 | 0 | 1 |
| *Rotundomys intimus* sp. nov. | 1 | 0 | 2 | 0 | 1 | 1&2 | 1 | 1 | 1&2 | 0&1 | 0&1 | 0 | 1 | 1 | 2 | 1&2 | 0&1 | 0&1 | 1 | 0 | 1 |

**TEXT S2 Character/taxon matrix used in the analysis of relationships of all species of *Cricetulodon* and *Rotundomys*. Characters are listed in Text S1. Character scoring: 0, 1 and 2, conditions of character; ?, character state uncertain.**

|  | 22 | 23 | 24 | 25 | 26 | 27 | 28 | 29 | 30 | 31 | 32 | 33 | 34 | 35 | 36 | 37 | 38 | 39 | 40 | 41 | 42 |
| --- | --- | --- | --- | --- | --- | --- | --- | --- | --- | --- | --- | --- | --- | --- | --- | --- | --- | --- | --- | --- | --- |
| *Democricetodon* | 1 | 0 | 2 | 0 | 0 | 0 | 0 | 0 | 0 | 0 | 0 | 0 | 0 | 0 | 0 | 0 | 0 | 0 | 1 | 1 | 0 |
| *Cricetulodon hartenbergeri* | 0 | 0 | 1&2 | 0 | 0 | 0 | 0 | 0 | 0 | 1 | 0 | 0 | 0 | 0&1 | 0 | 0 | 0 | 0 | 0 | 0 | 0 |
| *Cricetulodon sabadellensis* | 0&1 | 0 | 2 | 0 | 1 | 0 | 0 | 0 | 0&1 | 1 | 0 | 0 | 0 | 1 | 0 | 0 | 0 | 0 | 0 | 0 | 0 |
| *Cricetulodon bugesiensis* | 0&1 | 0 | 2 | 0 | 0 | 0 | 0 | 0 | 0&1 | 1 | 0 | 1 | 1 | 1 | 1 | 0 | 0 | 0 | 1 | 1 | 0 |
| *Cricetulodon meini* | 1 | 0 | 0 | 0 | 0 | 0 | 0 | 0 | 0 | 1 | 0 | 0 | 0 | 0&1 | 0 | 0 | ¿* | ¿ | ¿ | ¿ | ¿ |
| *Cricetulodon lucentensis* | 0&1 | 1 | 2 | 0&1 | 0 | 0 | 0 | 0 | 0 | 1 | 0 | 0 | 0 | 0&1 | 1 | 0 | 0 | 0 | 1 | 1 | 0 |
| *Rotundomys montisrotundi* | 1 | 0 | 2 | 0&1 | 1 | 1 | 2 | 1 | 1 | 2 | 0&1 | 1 | 1 | 1 | 1 | 0&1 | 1 | 1 | 1 | 1 | 0&1 |
| *Rotundomys bressanus* | 1 | 0 | 2 | 1 | 1 | 2 | 2 | 1 | 1 | 2 | 1 | 1 | 1 | 1 | 1 | 1 | 1 | 1 | 1 | 1 | 1 |
| *Rotundomys mundi* | 1 | 0 | 2 | ¿ | 1 | 1 | 2 | 0 | 1 | 2 | ¿ | 1 | 0 | 1 | 1 | ¿ | 1 | 1 | 1 | 1 | ¿ |
| *Rotundomys sabatieri* | 1 | 0 | 2 | 0&1 | 1 | 1 | 2 | 1 | 1 | 2 | 0&1 | 1 | 1 | 1 | 1 | 0 | 1 | 1 | 1 | 1 | 0&1 |
| *Rotundomys freiriensis* | 1 | 1 | 2 | ¿ | 1 | 1 | 0 | 0 | 1 | 3 | ¿ | 0 | 0 | 1 | 0 | ¿ | 1 | 1 | 1 | 1 | ¿ |
| *Rotundomys* sp.nov. | 1 | 0 | 2 | 0&1 | 1 | 1 | 1 | 1 | 1 | 2 | 0&1 | 1 | 1 | 1 | 1 | 0&1 | 1 | 1 | 1 | 1 | 0&1 |
