# Supplementary material for: New Species of Rotundomys (Cricetinae) from the Late Miocene of Spain and Its Bearing on the Phylogeny of Cricetulodon and Rotundomys
Source: PLoS One. 2014 Nov 12;9(11):e112704. doi: 10.1371/journal.pone.0112704 (PMC4229238; doi:10.1371/journal.pone.0112704)
Supplement: Text S3 — Analyses of the variance (ANOVA), including the Levene test for Homogeneity of Variances, for Length (L) and Width (W) of the Type material of Rotundomys freiriensis from Freiria do rio Maior, R. intimus nov. sp. from Batallones 5, R. sabatieri from Lo Fournas 16M, and R. mantisrotundi from Montredon. The last four columns indicate the homogeneous subsets calculated by Tukey's Post Hoc test (alpha = 0.05). (DOCX) [file pone.0112704.s003.docx]

| Element |  | Homogeneity of  Variances | | ANOVA | | *R. freiriensis* | *R. intimus* nov. sp. | *R. sabatieri* | *R. mantisrotundi* |
| --- | --- | --- | --- | --- | --- | --- | --- | --- | --- |
|  |  | Levene | Sign. | F | Sign. |  |  |  |  |
| M1 | L | 0.695 | 0.501 | 6.830 | 0.001 | - | a | b | b |
|  | W | 0.809 | 0.447 | 15.886 | <0.001 | - | a | b,c | c |
| M2 | L | 0.773 | 0.511 | 10.614 | <0.001 | a | b | b | b |
|  | W | 0.349 | 0.790 | 18.777 | <0.001 | a | a,b | b,c | c |
| M3 | L | 0.820 | 0.485 | 18.953 | <0.001 | a | b | b | b |
|  | W | 0.568 | 0.637 | 18.786 | <0.001 | a | b | b | b |
| m1 | L | 0.810 | 0,490 | 22.728 | <0.001 | a | b | b,c | c |
|  | W | 3.227 | 0,220 | 26.965 | <0.001 | a | b | b | b |
| m2 | L | 1.186 | 0.317 | 35.600 | <0.001 | a | b | b,c | c |
|  | W | 0.296 | 0.828 | 29.251 | <0.001 | a | b,c | b | c |
| m3 | L | 1.152 | 0.330 | 45.084 | <0.001 | a | b | c | c |
|  | W | 2.445 | 0.066 | 20.676 | <0.001 | a | b | b | b |

**TEXT S3 Analyses of the variance (ANOVA), including the Levene test for Homogeneity of Variances, for Length (L) and Width (W) of the Type material of *Rotundomys freiriensis*  from Freiria do rio Maior, *R. intimus* nov. sp. from Batallones 5, *R. sabatieri* from Lo Fournas 16M, and *R. mantisrotundi* from Montredon. The last four columns indicate the homogeneous subsets calculated by Tuckey’s Post Hoc test (alpha = 0.05).**
